# Gamified Learning in a Virtual World for Undergraduate Emergency Radiology Education: Quasi-Experimental Study

**DOI:** 10.2196/68518

**Published:** 2025-08-05

**Authors:** Alba Virtudes Pérez-Baena, Teodoro Rudolphi-Solero, Rocío Lorenzo-Álvarez, Miguel José Ruiz-Gómez, Francisco Sendra-Portero

**Affiliations:** 1 Department of Radiology, Hospital Comarcal de Antequera, Antequera, Spain; 2 Department of Radiology and Physical Medicine, Facultad de Medicina, Universidad de Málaga, Bvd. Luis Pasteur, 32., Málaga, 29071, Spain, 34 606266461; 3 Department of Emergency and Intensive Care, Hospital de la Axarquía, Vélez-Málaga, Spain

**Keywords:** radiology education, medical students, computer simulation, virtual worlds, emergency radiology, game-based learning, case-based learning

## Abstract

**Background:**

Emergency radiology is essential for future doctors, who will face urgent cases requiring radiologic diagnosis. Using virtual simulations, gamified clinical scenarios, and case-based learning enhances practical understanding, develops technical and communication skills, and fosters educational innovation.

**Objective:**

This study aimed to assess the feasibility of learning emergency radiology in the virtual world Second Life (Linden Lab) through a gamified experience by evaluating team performance in clinical case resolution, individual performance on seminar assessments, and students’ perceptions of the activity.

**Methods:**

Teams of 3‐4 final-year medical students, during a 2-week radiology clerkship, had access to 7 clinical cases in virtual clinical stations and were randomly assigned 2 to solve and submit. They later discussed the cases in a synchronous virtual meeting and attended an emergency radiology seminar. The experience was repeated over 2 consecutive years to assess reproducibility through comparison of learning outcomes and students’ perceptions. Learning outcomes were evaluated through team-based case resolution and individual seminar assessments. Students’ perceptions were gathered via a voluntary questionnaire including 5-point Likert scale items, cognitive load ratings, 10-point evaluations, and open-ended comments.

**Results:**

In total, 182 students participated in 2020‐2021 and 170 in 2021‐2022, demonstrating strong team-based case resolution skills with mean scores of 7.36 (SD 1.35) and 8.41 (SD 0.99), respectively (*P*<.001). The perception questionnaire had a 90.6% response rate. The highest cognitive load was observed in avatar editing (median 7, 95% CI 6.56‐6.96). Case-solving cognitive load was significantly lower in 2021‐2022 compared with 2020‐2021 (median 6, 95% CI 5.69‐6.21 vs 5.10‐5.66; *P*<.001). The students rated the experience highly, with average scores exceeding 8.0 out of 10 across various aspects. Notably, the highest-rated aspects were the teaching staff (9.13, SD 1.15), cases (8.60, SD 1.31), project organization (8.42, SD 1.67), and virtual rooms (8.36, SD 1.62). The lowest-rated aspect was internet connectivity (6.68, SD 2.53). Despite the positive scores, all aspects were rated significantly lower in 2021‐2022 compared with 2020‐2021. These year-to-year comparisons in performance and perception support the reproducibility of the experience.

**Conclusions:**

This study demonstrates that a game-based learning experience in the Second Life virtual world, combining virtual clinical scenarios and team-based tasks, is feasible and reproducible within a radiology clerkship. Students showed strong performance in case resolution and rated the experience highly, within a playful context that integrated asynchronous and synchronous activities. Lower ratings in the second year may reflect contextual differences, such as changes in COVID-19 pandemic restrictions.

## Introduction

Gamification is the use of game elements in nongame environments, such as in education. This alternative educational approach promotes student motivation and active participation in the learning process [1]. Its implementation has been shown to enhance engagement, increase motivation, improve knowledge retention, and develop problem-solving skills [2-4]. The effectiveness of gamification is rooted in the fulfillment of 3 basic psychological needs [2]: (1) autonomy—the need to feel in control of one’s actions; (2) competence—the desire to achieve goals and experience success [5]; and (3) relatedness—the need to feel connected to others and be part of a group [6]. Through game-based tasks, students are encouraged to experiment, interact, and collaborate with peers [7]. However, gamification alone is not sufficient to ensure meaningful learning outcomes. Its effectiveness depends on thoughtful design and alignment with the specific skills students are expected to acquire [8]. To support knowledge acquisition and consolidation, game elements must be carefully selected based on academic goals, student needs, and a pedagogically sound teaching methodology [9]. In this context, gamification proves to be a viable strategy at the university level—particularly in undergraduate programs—as a means to fulfill the educational goals outlined in academic curricula [10]. Studies have reported positive academic outcomes, high levels of student engagement, and strong adherence to gamified activities [11-14]. At the postgraduate level, gamification has also found applications in health care education. It has been used with surgical residents through the Da Vinci simulator to enhance surgical skills [15], with internal medicine residents—both individually and in teams—via online platforms to update clinical knowledge [16], with radiology residents through virtual tools for interpreting chest x-rays [17], and with otorhinolaryngology residents to assess and train new laryngoplasty techniques [18].

Game-based learning, often referred to as digital games [19,20], supports students in achieving learning objectives through immersive and engaging learning experiences. These activities have shown promising results with medical students, especially when implemented in immersive digital platforms [21,22], and their use in undergraduate education is worth exploring. These immersive platforms, known as virtual worlds, are computer-generated 3D spaces where people interact remotely through representations of themselves called avatars. The concept of virtual worlds, along with others such as virtual reality, mirror worlds, and augmented reality, fits within the broader notion of the metaverse [23], a collective virtual shared space expressed through digital media and the internet. Clinical simulation environments in such settings have been developed to train various skills, including taking anamnesis from virtual patients [24], resolving clinical situations in a pneumology ward [21], training cardiopulmonary resuscitation [25], or developing communication skills with patients [26,27].

One of the most widely used virtual worlds for health professional education is Second Life (SL; Linden Lab) [28]. Its advantages include remote access, a strong sense of presence, ease of access, user anonymity, opportunities to develop communication skills, promotion of active learning, and being free of charge. SL allows educators to design and recreate clinical training scenarios [29], including those based on the Objective Structured Clinical Examination (OSCE) format, in which specific “stations” simulate clinical cases in a standardized, reliable, and objective way [30]. It has already proven to be a valuable tool for teaching radiology in both synchronous and asynchronous online formats [31], and it has supported interactive learning focused on radiological anatomy and imaging signs [22]. However, to our knowledge, no experiences have yet incorporated radiology OSCE-like scenarios using gamified approaches in SL or similar environments.

Emergency radiology is essential for medical students, as future doctors will inevitably encounter urgent clinical scenarios requiring timely and accurate radiologic diagnosis. The ability to interpret commonly used x-ray examinations in the primary assessment of acutely unwell patients, and to recognize basic findings on emergency computed tomography (CT) scans, is considered a core competency in undergraduate medical training [32]. The European Society of Radiology highlights the importance of educating medical students in emergency radiology to ensure they understand imaging’s critical role in acute care and can contribute effectively in managing clinical emergencies [33]. Furthermore, teaching emergency radiology to medical students has been shown to enhance their knowledge, promote appropriate imaging usage, support evidence-based decision-making, and increase awareness of the potential downstream effects of incidental findings [34].

This study aimed to assess the feasibility of learning emergency radiology in the virtual world SL through a gamified experience by evaluating team performance in clinical case resolution, individual performance on seminar assessments, and students’ perceptions of the activity. The educational activity combined case-based problem-solving in OSCE-style stations with virtual online seminars. The activity was developed for sixth-year medical students (in their final year of medical school in our country) as part of a formal radiology clerkship.

## Methods

### Overview

This was a quasi-experimental study conducted over 2 academic years to evaluate a virtual, gamified radiology learning experience.

### Background and Project Design

Sixth-year medical students at our university complete a 2-week radiology clerkship, consisting of 4 days of hospital practice, ten 2-hour seminars on clinical radiology, and online learning activities. Each year, 7 groups of 24‐28 students successively complete the radiology clerkship between mid-October and early February. Eligibility criteria included enrollment in this clerkship during the 2020‐2021 or 2021‐2022 academic years. All students met these criteria, and no exclusions were applied. The educational purpose of the activity in this study, called “Rainbow-Game,” was to provide students with reflective training on clinical situations involving medical emergencies mediated by the corresponding radiological procedures, in a playful and collaborative context, as part of the activities of the clerkship.

Each group was randomly divided into 7 teams of 3‐4 students named with the colors of the rainbow. On the first day of the clerkship, students were briefed on the details of this experience. They had to dress their avatar in shirts of their team’s color and had 9 days to visit, on demand, a virtual space with 7 OSCE stations, each containing an emergency radiology case. Students had unrestricted access to all stations throughout this period, encouraging self-directed learning and flexibility in how they engaged with the cases. Although they were encouraged to review all 7 cases, only 2 were randomly assigned to each team on the eighth day for formal written resolution and submission via SL’s internal messaging system (notecard). To support them, they received a short orientation on how to use SL and step-by-step mini-tutorials to assist with independent navigation, creating and sending notecards, and customizing their avatars with the team T-shirts. Although students used their own devices, they were advised to ensure basic technical compatibility and stable internet access. As part of the game, teams also had to send the professor original and imaginative SL screenshots showing their avatars dressed in the corresponding color. On the tenth day, there was a 2-hour meeting with the whole group in SL. First, the teams had to orally present the results of the assigned cases and discuss them with their peers. Subsequently, they received a 1-hour seminar on emergency radiology, in which the professor presented 15 clinical cases, distributed equally among head, chest, and abdominal emergencies. At the end of the seminar, 3 exam cases were arranged to be answered individually using a notecard. A 24-item checklist was used to correct answers uniformly (see Multimedia Appendix 1). This activity was repeated during 2 academic years (2020‐2021 and 2021‐2022) with the same contents and organization. The TREND (Transparent Reporting of Evaluations with Nonrandomized Designs) statement [35] was followed to ensure transparent and comprehensive reporting.

### Outcomes and Assessment

The study evaluated three categories of outcomes: (1) team performance in clinical case resolution, (2) individual performance on seminar assessments, and (3) students’ perceptions of the activity. Team performance was scored using a structured checklist (0‐10 points per case), and individual seminar responses were graded using a 24-item checklist (Multimedia Appendix 1). In both cases, higher scores indicated better performance.

Students’ perceptions were captured through a questionnaire adapted from a previously validated instrument [22] (Multimedia Appendix 2) including 5-point Likert items (ordinal), 9-point cognitive load ratings (ordinal), 10-point satisfaction ratings (continuous), and open-ended comments. Higher Likert scores reflected more positive perceptions, higher cognitive load scores indicated greater perceived mental effort, and higher satisfaction ratings indicated greater satisfaction.

### Virtual Scenarios

**Figure 1.**
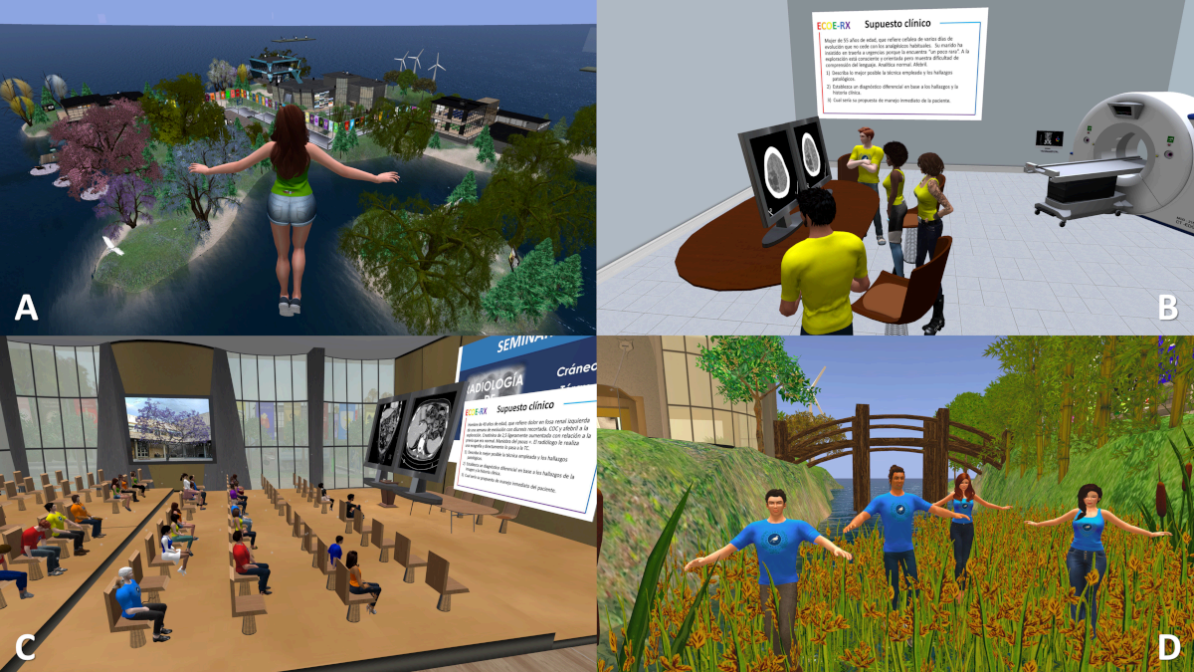
Various scenes during the Second Life virtual world experience. (A) Aerial view of Medical Master Island with a flying avatar in the foreground. In the background, the main building, where the synchronous seminars were held, can be seen. (B) Example of an Objective Structured Clinical Examination station set up as a radiology room, with a team of students reviewing a head computed tomography case. The description of the clinical situation and the tasks to be performed are displayed on the wall. (C) Scene during a synchronous meeting with a student group, reviewing one of the Objective Structured Clinical Examination cases. (D) Screenshot submitted by a team of students as part of the competition.

**Figure 2.**
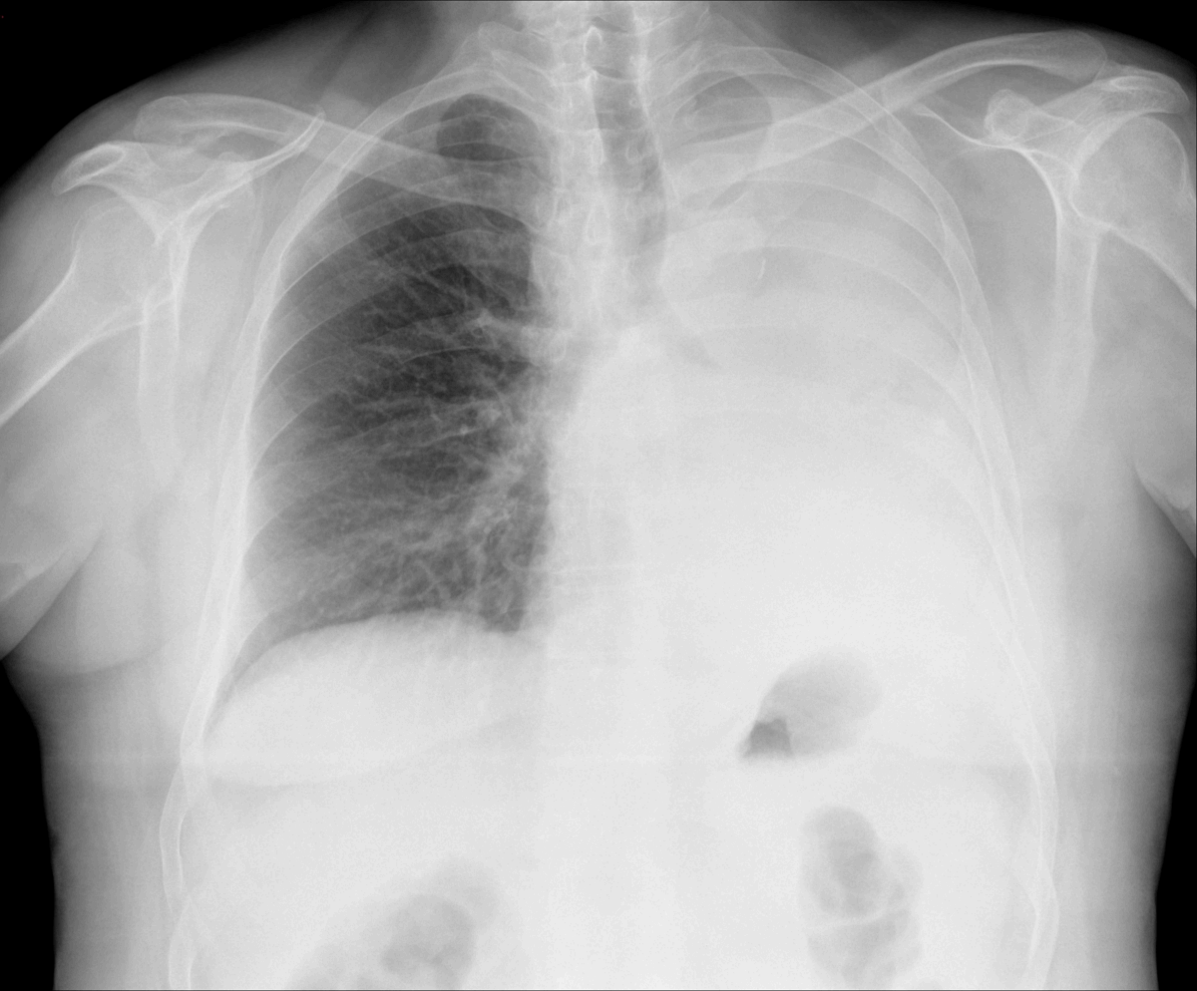
A chest x-ray corresponding to case number 3 shows complete opacification of the left hemithorax due to atelectasis of the left lung caused by bronchial carcinoma.

This study was conducted at the SL location named “The Medical Master Island,” designed with various academic buildings surrounded by trees and plants (Figure 1A). Seven OSCE stations were designed with access from the same distributor, imitating a real OSCE. Each station had: (1) an access door; (2) a panel on the wall describing the clinical situation and the questions to be answered; (3) a table with one or 2 monitors, showing the images of the case; and (4) x-ray or CT equipment to contextualize the place as a radiology room (Figure 1B). To minimize repetitions, 16 cases were used (Table 1), which were rotated for the 7 groups (Table 2). An example case is shown in Figure 2. The clinical situation presented to the students is that of a 65-year-old woman who went to the emergency department due to progressive dyspnea occurring with minimal effort, reporting weight loss over the last month. Her personal history includes smoking and dyslipidemia. On examination, she is conscious, oriented, and cooperative, with mild respiratory retractions. Global hypoventilation in the left lung is noted. Oxygen saturation is 85%. She is afebrile. An imaging test is performed. Tasks to be carried out included describing as accurately as possible the imaging technique used and the pathological findings, establishing a differential diagnosis, and outlining the clinical approach to the suspected condition. Indicate what other tests would be required and why. The group meeting was held in the main building’s aula magna, which had monitors with images to discuss the cases and a panel of slides to present the seminar (Figure 1C). Students submitted screenshots of their teams as part of the competition (Figure 1D).

**Table 1.** Description of the 16 cases used in the Rainbow-Game.

Case	Image modality shown	Case description
1	Brain CT^a^ without IV^b^ contrast: 18 axial slices.	52-year-old man. Spontaneous intraparenchymal hemorrhage with a subarachnoid component.
2	Abdominal CT with IV contrast: 18 axial slices and 18 coronal slices.	39-year-old woman. Multiple renal lacerations with hemoperitoneum.
3	Chest x-ray: posteroanterior.	65-year-old woman. Complete opacification of the left hemithorax: atelectasis of left lung due to a bronchial carcinoma.
4	Brain CT without IV contrast: 18 axial slices.	79-year-old man. Acute ischemic lesion in the left cerebral hemisphere: MCA stroke with subfalcine herniation.
5	Abdominal radiography: oblique.	73-year-old man. Large bowel dilatation with coffee bean sign: Acute sigmoid volvulus with pneumoperitoneum.
6	Abdominal CT with IV contrast: 18 axial slices.	55-year-old woman. Gallbladder distention, wall thickening, mucosal hyperenhancement, and pericholecystic fat stranding: Acute cholecystitis.
7	Brain CT without IV contrast: 18 axial slices plus 18 in bone window.	40-year-old man. Parietal acute epidural hematoma with associated bone fracture.
8	Chest x-ray: posteroanterior.	59-year-old woman. Widening of the mediastinum, wide aortic contour, tracheal deviation, aortic kinking: Acute aortic dissection.
9	Abdominal CT with IV contrast: 18 axial slices.	55-year-old man. Ruptured abdominal aortic aneurysm with hemoperitoneum.
10	Brain CT without IV contrast: 18 axial slices plus 18 in bone window.	58-year-old man. Frontal intraparenchymal hemorrhage, subdural hemorrhage, and occipital skull fracture.
11	Chest x-ray: posteroanterior.	65-year-old man. Pulmonary consolidation without volume loss, air bronchogram, and silhouette sign: Lobar pneumonia.
12	Abdominal CT with IV contrast: 18 axial slices.	60-year-old man. Colonic wall thickening, pericolic fat stranding in an area of sigmoid diverticulosis: Acute diverticulitis.
13	Chest x-ray: posteroanterior.	62-year-old man. Consolidations and ground-glass opacities bilateral, peripheral, and located in the lower fields: COVID-19 pneumonia.
14	Brain CT with and without IV contrast: 2 sets of 18 axial slices.	55-year-old woman. Single cortical lesion, round, well-demarcated with enhancement and perilesional vasogenic edema: Metastatic lesion.
15	Abdominal CT with IV contrast: 18 axial slices and 18 coronal slices.	49-year-old man. Large soft-tissue mass, with internal heterogeneity: Retroperitoneal sarcoma.
16	Chest x-ray: posteroanterior and lateral.	76-year-old man. Bone osteoblastic lesions: Metastasis due to prostate carcinoma.

a CT: computed tomography

b IV: intravenous.

**Table 2.** Assignment of cases to the Objective Structured Clinical Examination stations across different groups.

Groups^a^	Station 1	Station 2	Station 3	Station 4	Station 5	Station 6	Station 7
Group 1	Case 1	Case 2	Case 3	Case 4	Case 5	Case 6	Case 7
Group 2	Case 8	Case 9	Case 10	Case 11	Case 12	Case 13	Case 14
Group 3	Case 15	Case 16	Case 1	Case 2	Case 3	Case 4	Case 5
Group 4	Case 6	Case 7	Case 8	Case 9	Case 10	Case 11	Case 12
Group 5	Case 13	Case 14	Case 15	Case 16	Case 1	Case 2	Case 3
Group 6	Case 4	Case 5	Case 6	Case 7	Case 8	Case 9	Case 10
Group 7	Case 11	Case 12	Case 13	Case 14	Case 15	Case 16	Case 1

a The same cases were used for the 7 consecutive groups of students in both the 2020–2021 and 2021–2022 academic years.

### Qualification of the Participants

Each team was qualified based on 3 parameters, normalized to 10 points each: (1) the written response to the 2 assigned cases, using a checklist for each case (40%); (2) the originality and quality of the best screenshot submitted (20%); and (3) the average of the individual points of the team to the 3 seminar questions (40%). Students were informed that their team’s score on this compulsory online activity would have no impact on their clerkship grades. However, as extrinsic motivation, the team with the highest score in each group received a bonus of one extra point in the final grade, up to 10 points.

### Perception of the Experience by the Students

After the seminar, the students were asked to complete a questionnaire about the experience (see Multimedia Appendix 2), which included: (1) a dichotomous question about whether they knew SL before the experience; (2) an assessment of 13 aspects of the game with a Likert scale of 1‐5 (from totally disagree to totally agree); (3) an assessment of the cognitive load of 6 aspects of the game, following the 9-point scale proposed by Paas and Merriënboer [36]; (4) a rating of up to 10 points on 9 aspects of the game; and (5) a space for open comments asking “anything else to add.” The questionnaire contained questions constructed, worded, and validated in previous studies [22,31].

### Data Analysis

Descriptive statistics were performed using Microsoft Excel 2021 to characterize the population and subpopulations of participants, and the SPSS statistical package v24 (IBM Corporation) was used for statistical analysis. The normality of the data was assessed using the Shapiro–Wilk test. Based on the distribution of each variable, unpaired 2-sample *t* tests were used for continuous variables that met the normality assumption, while Mann–Whitney *U* tests were applied to compare ordinal or nonnormally distributed variables. Statistical significance was accepted at a probability of error of *P*<.05. Reproducibility was assessed by comparing student performance (project scores and seminar results) and perception data (questionnaire responses) across the 2 academic years.

Open comments were analyzed using the systematic collaborative consensus coding by committee [37]. Comments related to aspects of the clerkship other than the Rainbow-Game were not considered. During 2 consensus meetings, a 2-layer hierarchical coding was established, with 3 codes in the first layer (advantages, disadvantages, and suggestions) and different subcodes in the second layer.

### Ethical Considerations

This study received approval from the Institutional Ethics Committee for Experimentation at the University of Malaga (decision number 141‐2022-H; approval date: January 18, 2023). Questionnaire participation was voluntary, and participants gave their explicit informed consent at the time of submission. All data collected were anonymized before analysis to ensure the privacy and confidentiality of participants. Students were informed that their participation would not affect their academic evaluation and had the opportunity to opt out without any consequences. No monetary or material compensation was provided for participation in the study.

## Results

### Outcome of the Rainbow-Game

Three hundred and fifty-two students participated in this project, 182 in 2020‐2021 and 170 in 2021‐2022. Of the 352 students, 227 (64.5%) were women and 125 (35.5%) were men. The mean age was 23.7  ( SD 2.8) years, with a median of 23 and a range from 22 to 51 years.

**Figure 3.**
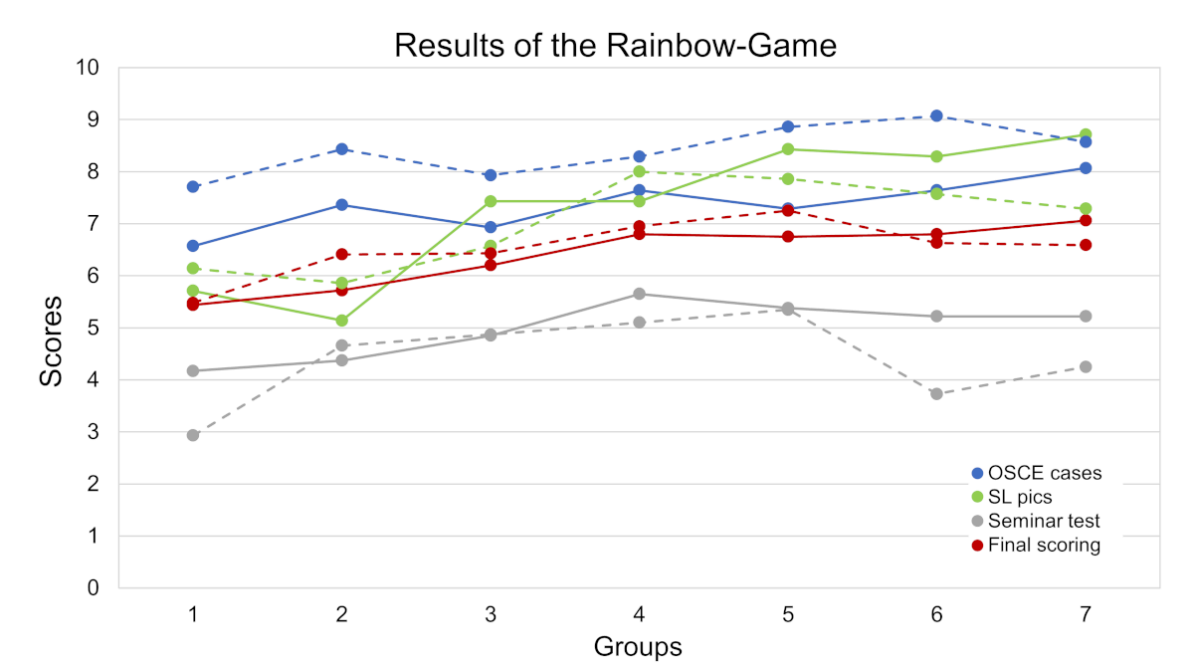
Scoring of the different components of the Rainbow-Game educational experience. OSCE: Objective Structured Clinical Examination; SL pics: Second Life pictures.

The normality of the distributions was assessed using the Shapiro–Wilk test. OSCE case scores (2020: W=0.934, *P*=.009; 2021: W=0.891, *P*<.001) and the final experience score (2020: W=0.874, *P*<.001; 2021: W=0.944, *P*=.02) did not follow a normal distribution and were compared using the Mann–Whitney *U* test. In contrast, the results of the seminar questions (2020: W=0.966, *P*=.17; 2021: W=0.981, *P*=.59) and academic clerkship grades (2020: W=0.968, *P*=.20; 2021: W=0.967, *P*=.18) followed a normal distribution and were compared using unpaired 2-sample *t* tests. The average rating up to 10 points of the teams in 2020‐2021 versus 2021‐2022 was as follows: in OSCE cases, 7.36 (SD 1.35) versus 8.41 (SD 0.99*; P*<.001); screenshots taken in SL, 7.31 (SD 1.65) versus 7.04 (SD 1.73; *P*=.35); the seminar questions 4.98 (SD 0.83) versus 4.41 (SD 1.07; *P*=.004); and the final score of the experience, 6.40 (SD 0.86) versus 6.54 (SD 0.77;* P*=.40). Figure 3 shows the average score of the 7 groups for each year in chronological order. There was no difference in the academic grade of the clerkship between the students of both years (7.68, SD 0.44 vs 7.79, SD 0.43; *P*=.19).

**Figure 4.**
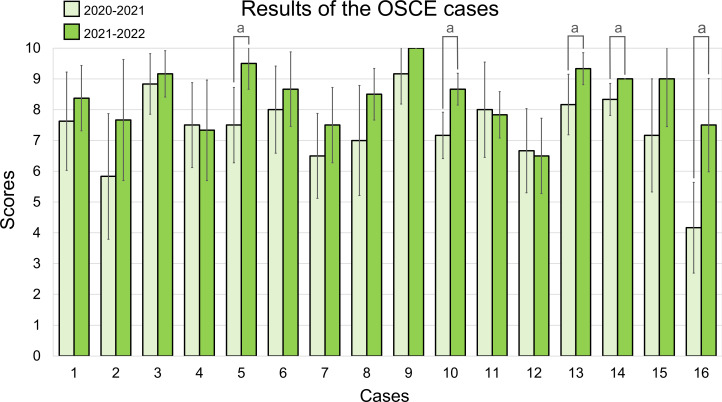
Bar chart with the average score obtained for each case over 2 consecutive years. Each year, 6 teams evaluated the cases, except for case 1, which was evaluated by 8 teams. Error bars represent the SD. Statistically significant differences, with *P*<.05, are identified with the letter “a.” OSCE: Objective Structured Clinical Examination;

In Figure 3, the final scores were obtained by considering the percentage contribution of the OSCE cases (40%), the SL screenshots (20%), and the seminar test (40%). The points represent the mean values of the 7 teams in each group. The solid line corresponds to the 2020‐2021 academic year, and the dashed line to 2021‐2022. The average score obtained in the 16 OSCE cases is shown in Figure 4. In 5 cases, the score obtained in 2021‐2022 was significantly higher than in 2020‐2021; there were no other significant differences. Considering both years, the easiest case was number 9, a ruptured abdominal aortic aneurysm with hemoperitoneum on CT (mean score 9.58, SD 0.79), and the most difficult was number 16, osteoblastic bone metastases from prostate carcinoma in a chest x-ray (5.83, SD 2.25).

### Students’ Perception

Three hundred and nineteen students (90.6%) submitted the perception questionnaire, 167 (91.8%) in 2020‐2021 and 152 (89.4%) in 2021‐2022. Only 38 (11.9%) stated that they did not know SL previously, the rest had participated in educational activities in SL during their third year. All items from the questionnaire showed significant deviation from normality, with Shapiro–Wilk tests yielding *P*<.001 in both cohorts. Consequently, all comparisons were performed using the Mann–Whitney *U* test.

The results regarding cognitive load are summarized in Table 3. The task that required the greatest mental effort was editing and dressing the avatar, with an average score between rather high and high mental effort (median 7, 95% CI 6.56‐6.96), followed by solving the cases proposed in the OSCE stations, with an average score between no mental effort and rather high mental effort (median 6, 95% CI 5.48‐5.86). The rest of the tasks required from rather low to very low mental effort. There were no significant differences in cognitive load between the 2 years except for the tasks of dressing the avatar, which required greater mental effort in 2021‐2022, and the resolution of OSCE cases, which required less mental effort in 2021‐2022.

**Table 3.** Results of the questionnaire about the cognitive load.

How much mental effort does it cost you to develop the following tasks?^a^	2020‐2021			2021‐2022			*P* value^b^	Both years		
	Mean (SD)	Median (95% CI)		Mean (SD)	Median (95% CI)			Mean (SD)	Median (95% CI)	
Moving around in Second Life	3.85 (2.25)	4 (3.51‐4.19)		3.93 (2.25)	3 (3.57‐4.29)		.59	3.89 (2.25)	3 (3.64‐4.14)	
Communicate by written chat	2.07 (1.52)	2 (1.84‐2.30)		2.23 (1.69)	2 (1.96‐2.50)		.50	2.15 (1.60)	2 (1.97‐2.33)	
Communicate by voice	2.67 (2.14)	2 (2.34‐3.00)		2.99 (2.34)	2 (2.62‐3.36)		.19	2.83 (2.24)	2 (2.58‐3.08)	
Edit and dress your avatar	6.62 (1.81)	7 (6.34‐6.90)		6.90 (1.85)	7 (6.61‐7.19)		.08	6.76 (1.83)	7 (6.56‐6.96)	
Solve the proposed cases in the OSCE-RX^c^ room	5.95 (1.71)	6 (5.69‐6.21)		5.38 (1.73)	6 (5.10‐5.66)		<.001	5.67 (1.74)	6 (5.48‐5.86)	
Follow the development of the seminar in Second Life	4.54 (2.26)	5 (4.19‐4.89)		4.44 (1.92)	5 (4.14‐4.74)		.33	4.49 (2.10)	5 (4.26‐4.72)	

a Likert scale from 1 to 9 according to: (1) Very, very low mental effort; (2) Very low mental effort; (3) Low mental effort; (4) Rather low mental effort; (5) Neither high nor low mental effort; (6) Rather high mental effort; (7) High mental effort; (8) Very high mental effort; and (9) Very, very high mental effort.

b *P* is the probability of error of the Mann Whitney *U* test. Statisitical significance set at *P*<.05.

c OSCE-RX: Objective Structured Clinical Examination: Radiology.

**Figure 5.**
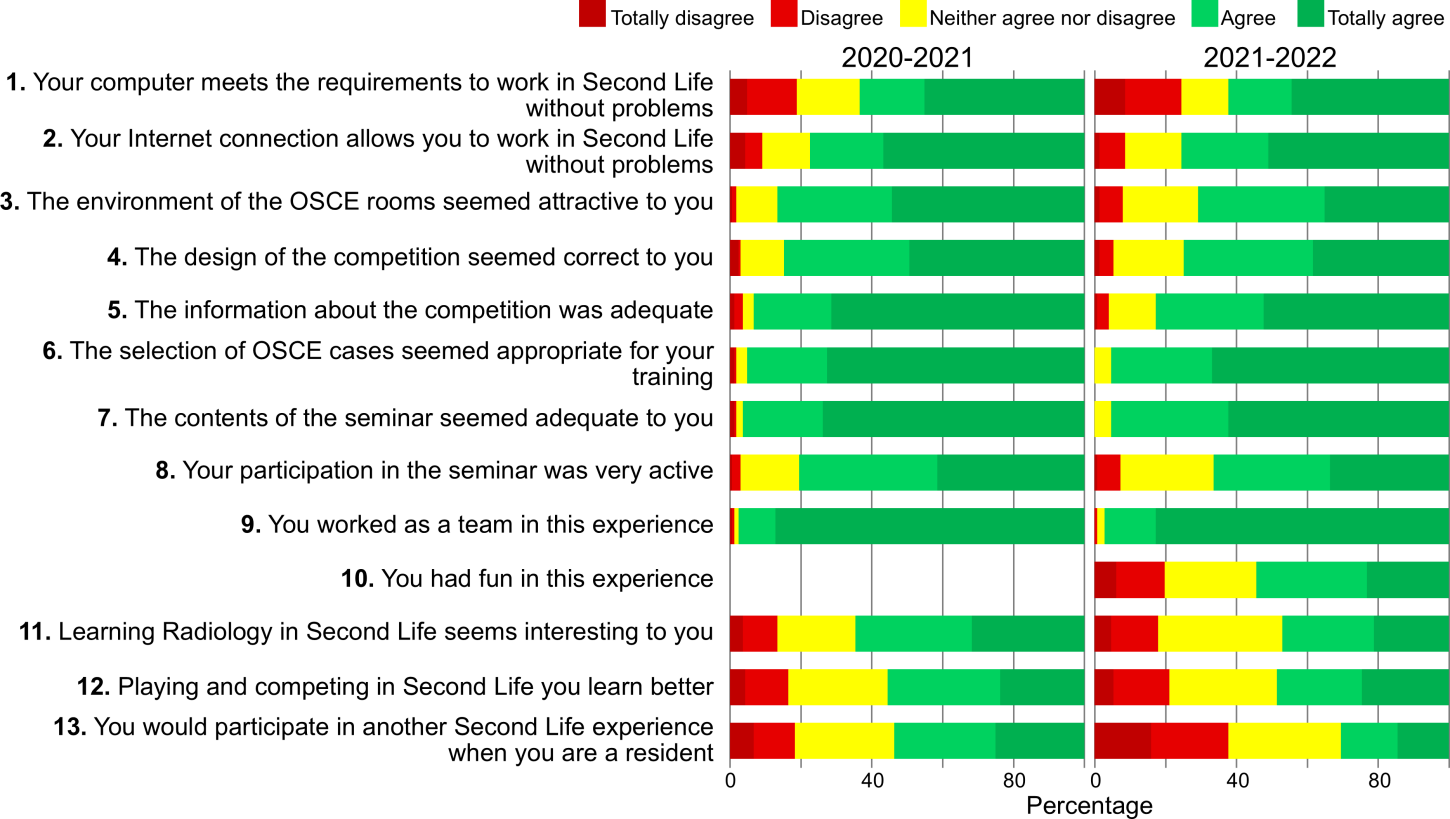
Diagram showing the degree of agreement, expressed as a percentage, reached each year in the responses to the experience evaluation questionnaire, which used a 1‐5 Likert scale. OSCE: Objective Structured Clinical Examination;

Overall, more than 95% of the respondents agreed or strongly agreed that the OSCE case selection was suitable for their training, the contents were appropriate, and that they worked as a team. In addition, more than 79% found the environment of the OSCE rooms attractive, the competition design appropriate, and the information provided adequate (Figure 5). Between 49% and 56% agreed that learning radiology in SL is interesting and that playing and competing is a better way to learn. Twenty-two percent of students disagreed with the suitability of their computers, and 9% disagreed with the adequacy of their internet connection for working in SL. Fifty-four percent of the 2021‐2022 students agreed that they had fun during the experience. There was significantly lower agreement in 2021‐2022 on 8 of the 5-point Likert scale statements.

The participants rated the experience up to 10 points, with mean scores higher than 8 points in 7 of 9 items (Table 4). The lowest rating was given for connectivity to SL. All mean scores in 2021‐2022 were significantly lower than those in 2020‐2021. One hundred and forty-three questionnaires (44.8%) included open comments: 82 (49.1%) in 2020‐2021, and 61 (40.1%) in 2021‐2022. After the initial first-layer coding, 12 second-layer subcodes were found among the advantages, 8 among the disadvantages, and 9 among the suggestions, as shown in Table 5, along with the thematic description and frequency.

**Table 4.** Rating in a 0‐10 points scale of various aspects of the experience.

Items	2020‐2021, mean (SD)	2021‐2022, mean (SD)	*P* value^a^	Both years, mean (SD)
Overall experience	7.81 (1.70)	7.07 (1.72)	<.001	7.46 (1.75)
Organization of the project	8,88 (1.45)	7.92 (1.75)	<.001	8.42 (1.67)
Environment of the OSCE^b^ rooms	8.66 (1.44)	8.03 (1.74)	<.001	8.36 (1.62)
OSCE cases	8.75 (1.35)	8.43 (1.25)	.007	8.60 (1.31)
The virtual seminar	8.63 (1.66)	7.62 (2.03)	<.001	8.15 (1.91)
The teachers	9.30 (1.17)	8.93 (1.10)	<.001	9.13 (1.15)
The utility for your training	8.52 (1.59)	7.56 (1.98)	<.001	8.06 (1.85)
Interaction with peers	8.55 (1.65)	7.73 (1.99)	<.001	8.16 (1.86)
Connectivity to Second Life	7.22 (2.37)	6.09 (2.57)	<.001	6.68 (2.53)

a *P* is the probability of error of the Mann–Whitney *U* tests data. Statistical significance set at *P*<.05.

b OSCE: Objective Structured Clinical Examination

**Table 5.** Thematic codification of the open comments included in the questionnaire.

Codes and subcodes		2020‐2021	2021‐2022	Both
Advantages				
Appreciation: with terms like I liked, interesting, attractive, gratifying, enjoyable, positive, very cool, fantastic.		30	19	49
Acknowledgment: recognition, thanks to teachers for the effort, design, and organization.		20	9	29
Didactic: indicating that the experience is useful for learning, profitable, helpful, formative.		14	11	25
Playful: fun, entertaining, expressing that you learn by playing.		18	9	27
Innovative: also expressed with terms such as new, original, unusual, surprising, creative, different.		13	8	21
Teamwork: highlighting its importance, social contact, collaboration, coworking.		14	4	18
Cases: finding them interesting, of balanced difficulty, useful or of didactic value for active learning.		6	9	17
Seminar: emphasizing the interest in medical training, the educational value or the feeling of presence and dynamics as in the classroom.		11	4	15
COVID: a good solution or adequate for the pandemic situation, which allows them to maintain social contact.		7	3	10
T-shirt: together with the picture contest, expressing that they liked it, thought it was a creative idea, or that it favors a good atmosphere.		3	1	4
Guidelines: highlighting that they were good, useful, or detailed.		4	0	4
2D Platforms: preference over 2D platform platforms such as Zoom, Google Meet, Microsoft Teams, etc.		0	2	2
Disadvantages				
Technical problems: due to the computer or the Internet connection, the program does not run well.		22	20	42
Mild or occasional		15	15	30
Serious (prevents executing SL)^a^		7	5	12
Of them, resolved		6	4	10
Second Life (SL): running SL is complicated, even stressful. They feel that they are not used to it or that they do not handle the interface well. A learning curve is necessary		13	11	24
Dressing up: the tasks of dressing the avatar are difficult, complicated, time-consuming, or not important to them.		8	9	17
Time: this activity becomes more complicated in the last year of the degree, in which there is a lot of occupation with other activities and tasks.		1	15	16
Face-to-face: preference for face-to-face seminars in the real world.		5	6	11
Camera: problems using the camera (avatar vision) and seeing the images properly.		6	4	10
Notecards: problems sending notecards		0	2	2
2D Platforms: preference for 2D online platforms, such as Zoom, Google Meet, Microsoft Teams, etc.		1	1	2
Suggestions				
Voluntary: proposal that these activities be voluntary, even on vacation.		0	7	7
Other platforms: to be used as a resource when Second Life fails or there are connection problems.		3	1	4
More cases: include more cases, with more modalities, such as ultrasound, breast imaging, etc.		2	1	3
Training SL: provide training on handling SL in medical school, including tricks, handling avatars, etc.		2	2	4
Modifications: slight modifications to the current experience related to scheduling, team building, or information		2	2	4
SL proposals: new gamification proposals, learning strategies, and radiology repositories. Even do all the clerkship seminars in SL.		4	0	4
Patients: add virtual patients to OSCE stations		1	0	1
Computers SL: enable computers with the SL viewer in the Faculty of Medicine.		0	1	1
Ubiquity: carry out the experience with professors and students from other universities.		0	1	1

a SL: Second Life

## Discussion

### Principal Findings

Today’s radiology students have different learning styles compared with previous generations, preferring to shift from the traditional lecture-based approach to more active learning methods that engage them more effectively [38]. This study presents a new educational experience for sixth-year medical students, conducted in SL during a 2-week radiology clerkship and replicated over 2 academic years. This educational experience is grounded in a constructivist and experiential learning approach [39], integrating several complementary pedagogical strategies. These include game-based learning to foster engagement and motivation [40]; case-based learning to stimulate clinical reasoning [38]; and team-based learning to promote collaboration and peer discussion [41]. The design also combines asynchronous and synchronous components to support flexibility and self-regulated learning. Simulation-based activities in a virtual world (SL) provide a safe, immersive environment for clinical skill development [42,43]. In addition, the experience draws on Self-Determination Theory [44], addressing both intrinsic motivation (interest and enjoyment) and extrinsic motivation (competition and rewards). Further pedagogical framing may be informed by Bers’ Coding as Another Language approach [45], which conceptualizes digital environments as spaces for exploration, communication, and meaning-making—aligning with our use of SL for collaborative and immersive clinical learning.

Case-based learning connects theoretical knowledge with the clinical environment and encourages students to think like doctors. By reflecting on radiological images through clinical cases, students can appreciate the role of radiology in patient care [38]. The 24/7 availability of the content in SL enables the organization of asynchronous tasks, such as the 9-day period for evaluating OSCE cases in this study, adapting to students’ study schedules, an essential factor for reflection and self-regulated learning [46]. Team-based learning is particularly suitable for visual topics like radiology, as it promotes and facilitates group discussion of complex, real-life radiological cases [47]. Teamwork dynamics are vital in undergraduate medical training, as they foster collaboration among students with varying levels of knowledge and experience [4,48].

Virtual world technologies, such as SL, make avatar-mediated student assessment in simulated 3D scenarios technically feasible to set up and run. This has been demonstrated in home accident scenarios in geriatric medicine [49], as well as in office or hospital settings [27], but has never been used to replicate scenarios for learning radiology. In this study, 7 OSCE stations were developed to simulate clinical scenarios involving medical emergencies, providing students with a variety of cases. The environment created allowed students to work in groups remotely at significantly lower cost than a similar scenario in the real world.

The educational activity in this study includes a significant component of competitive team gamification, a cooperative learning technique that enhances students’ motivation and focus on learning tasks [48], combining group rewards with individual responsibility [50]. According to self-determination theory, motivation has 2 components [51,52]: intrinsic motivation, defined as participation in an activity because it is found to be inherently interesting and enjoyable, and extrinsic motivation, where participation is driven by external factors such as rewards, promotions, or the avoidance of academic failure. Competition is a powerful extrinsic motivator; however, it is criticized for creating high-pressure environments that reduce intrinsic motivation and hinder optimal learning [53]. Previous studies have shown that multi-user competitive games developed in SL through asynchronous activities can enhance medical students’ learning of basic radiological content, such as anatomy and semiology, and that students find these games highly beneficial for their training as physicians, both when competing individually [25,54] and in teams [55]. This study proposes a different approach to learning games, incorporating clinical content commonly found in medical practice, centered on case resolution through teamwork and supplemented by synchronous discussion and debate activities. In both courses, students recognized and appreciated the educational value of the Rainbow-Game experience.

SL is an appropriate environment to develop oral communication skills in clinical radiology through the presentation and discussion of content [56]. In this study, effective communication was fostered through group discussion and reflection on the cases. The experience concluded with a seminar on emergency radiology to reinforce students’ knowledge and clinical reasoning. Seminars conducted in SL provide a strong sense of co-presence and have an educational impact comparable to those held in person, as long as the same objectives, content, and script are maintained [57].

Emergency radiology is an essential part of the undergraduate medical curriculum, ensuring that students can recognize major radiological emergencies [58]. In this study, students delved into clinical reasoning on cases commonly found in hospital emergency settings, achieving good results on the cases assigned to each team, with differences mainly related to the difficulty of the cases (Figure 4). This provides a measure of student performance on clinical cases in the final year of medical school. In contrast, the seminar exam questions were scored poorly due to limited response time and possibly an excessive number of items on the assessment checklist (see Multimedia Appendix 1). Students in 2021‐2022 performed better on the cases than those in 2020‐2021 and reported a lower cognitive load to solve them. This could indicate that they were better prepared for this type of tasks; however, they performed worse on the seminar questions, and there were no significant differences in the academic grades for the clerkship, so some leakage of results from one year to another cannot be ruled out. Although no traditional learning group was included, similar clerkship grades across both years suggest that the SL-based activity maintained academic performance while adding value through engagement and teamwork.

In general, the students perceived the experience as innovative and appropriate for their training. They valued the selected cases, the design and organization of the project, and the opportunity to work collaboratively in teams. In their open comments, they frequently described the activity as “fun,” “interesting,” and “useful for learning,” highlighting its originality and the engaging nature of solving clinical cases in a virtual world. Several students also appreciated the social interaction and teamwork it encouraged, particularly under pandemic restrictions. However, some disadvantages were noted, particularly technical issues related to SL, such as interface difficulties or connectivity problems. Although students received technical support, using personal devices may have contributed to differences in their experiences. These issues may be related to the technical requirements of the platform, which must be installed locally and require specific hardware and internet conditions [59]. One student suggested enabling access to university computers with the SL viewer. The task of customizing avatars was often described as time-consuming and lacking clear educational purpose. A number of students, particularly in 2021‐2022, felt the activity required too much time, and some suggested it should be optional or scheduled during a less demanding academic period.

These differences in perception may be attributed to the timing of both experiences during different phases of the COVID-19 pandemic. In the first semester of 2020‐2021, students faced a stressful situation with restricted in-person academic activities, where online solutions were seen as essential for teaching. One year later, teaching had partially normalized, with some precautions still in place, but there was a certain sense of fatigue from the overuse of online activities [60]. This may have contributed to a greater perception of workload among the students and, consequently, a lower appreciation of the learning experience. Despite lower satisfaction scores in 2021‐2022, students performed better on case resolution tasks and reported lower cognitive load. This suggests that learning outcomes do not always align with perceived satisfaction. Improved performance may reflect greater digital familiarity and self-regulation skills developed through previous remote learning. In contrast, lower ratings may have been influenced by reduced novelty, increased academic pressure, or persistent fatigue from extended online education, rather than the activity’s educational value.

### Comparison With Previous Work

This type of learning experience may be less appealing to those who are not drawn to gamification and technology. In fact, it has been shown that mandatory participation in gamification activities in SL can lead to a lower perception and acceptance of the game by a proportion of students [54]. Mandatory activities contribute to the extrinsic motivation of medical students, but “imposed” gamification has a counterproductive effect, described as “mandatory fun” [61]. User acceptance of 3D virtual world technology is positively influenced by their perception of the ease of use, usefulness, enjoyment, and visual appeal of a virtual learning environment, which significantly impacts student satisfaction, learning outcomes, and retention [62,63]. Most participants in the Rainbow-Game found the experience interesting and suitable for learning, and more than half found it fun. However, some students may not have perceived sufficient reward in a compulsory learning game. Therefore, the option for the best team in each group to earn an additional point on their academic grade compensates for this by providing an extrinsic motivation.

This study demonstrates how a mandatory game-based learning experience in SL can be added to the formal teaching organization of a radiology clerkship. Training in case resolution, peer discussion, and attending a seminar on emergency radiology provides added value to the educational objectives of this clerkship in the final year of the degree. The design of the cases and OSCE stations, along with the positive perception of students, is another added value. The immediate continuation of this project, which has already been completed and is pending publication, involved the development of individual, synchronous radiology OSCEs conducted over a restricted period of several minutes per station, emulating face-to-face OSCEs, like those carried out at the end of the degree [64]. Future developments will focus on comparing the virtual world activity with a control group performing identical tasks either in real-world settings or in 2D virtual environments. This will allow for a more comprehensive evaluation of the added value of immersive 3D learning environments in radiology education.

### Limitations

The main limitation of this experience, consistent with previous studies [31,57], was the presence of technical problems with the computers and the online connection needed to run SL correctly, as recognized in the open comments of 13% of the questionnaires. However, two-thirds of these issues were minor or occasional problems, and a third were resolved through simple solutions, such as borrowing a computer or using a cable connection. Since SL is developed as computer software, it requires suitable hardware that meets minimum requirements. In addition, SL is an internet-based technology with a client/server structure, which relies on stable internet connectivity. Despite the significant increase in internet access for higher education students, many still face restricted connectivity [63]. Another limitation to consider is that this study was conducted at a single institution. Although the experience has been replicated over 2 academic years, it would be valuable to see if it could be implemented elsewhere, which would require the cooperation of professors from other institutions and the inclusion of the project in the teaching guide for the corresponding courses.

### Conclusions

This study presents a game-based radiology learning experience conducted in the SL virtual world, integrating simulated case-based learning in virtual OSCE stations, team-based competitive learning, and synchronous group sessions. The experience, adapted to a radiology clerkship, is feasible and reproducible, promoting clinical reasoning and teamwork among students in a playful context that they recognize and value highly. In addition, it is worth exploring the educational potential of OSCEs in 3D virtual environments for radiology and other medical disciplines.

## Supplementary material

10.2196/68518 Multimedia Appendix 1 Presentation of the 16 clinical cases and the seminar exam cases, along with the checklist used for their correction.

10.2196/68518 Multimedia Appendix 2 Perception questionnaire on the Rainbow-Game experience.

## Abbreviations

CT: computed tomography
OSCE: Objective Structured Clinical Examination
SL: Second Life
TREND: Transparent Reporting of Evaluations with Nonrandomized Designs

## References

[1] Aguado-Linares P, Sendra-Portero F (2023). Gamification: basic concepts and applications in radiology. Radiologia (Engl Ed).

[2] Van Roy R, Zaman B, Reiners T, Wood L (2014). Gamification in Education and Business.

[3] Barata G, Gama S, Jorge J, Gonçalves D (2015). Gamification for smarter learning: tales from the trenches. Smart Learn Environ.

[4] Dichev C, Dicheva D (2017). Gamifying education: what is known, what is believed and what remains uncertain: a critical review. Int J Educ Technol High Educ.

[5] Brigham TJ (2015). An Introduction to gamification: adding game elements for engagement. Med Ref Serv Q.

[6] Hense J, Klevers M, Sailer M, Horenburg T, Mandl H, Günthner W, Meijer SA, Smeds R (2014). Frontiers in Gaming Simulation.

[7] Teixes Argilés F (2014). Gamificación: Motivar Jugando.

[8] Cuadros L, López A (2020). Gamificación como estrategia para fortalecer la producción textual en Ciencias Naturales [Article in Spanish]. Rev Doc Univ.

[9] Mero J, Campuzano J, López S, Jara CH (2022). La gamificación como estrategia para la estimulación del aprendizaje de las ciencias naturales [Article in Spanish]. Polo Conoc.

[10] Rutledge C, Walsh CM, Swinger N (2018). Gamification in action: theoretical and practical considerations for medical educators. Acad Med.

[11] Bochennek K, Wittekindt B, Zimmermann SY, Klingebiel T (2007). More than mere games: a review of card and board games for medical education. Med Teach.

[12] Akl EA, Pretorius RW, Sackett K (2010). The effect of educational games on medical students’ learning outcomes: a systematic review: BEME Guide No 14. Med Teach.

[13] Akl EA, Sackett KM, Erdley WS (2013). Educational games for health professionals. Cochrane Database Syst Rev.

[14] Janssen A, Shaw T, Goodyear P, Kerfoot BP, Bryce D (2015). A little healthy competition: using mixed methods to pilot a team-based digital game for boosting medical student engagement with anatomy and histology content. BMC Med Educ.

[15] Kerfoot BP, Kissane N (2014). The use of gamification to boost residents’ engagement in simulation training. JAMA Surg.

[16] Nevin CR, Westfall AO, Rodriguez JM (2014). Gamification as a tool for enhancing graduate medical education. Postgrad Med J.

[17] Chen PH, Roth H, Galperin-Aizenberg M, Ruutiainen AT, Gefter W, Cook TS (2017). Improving abnormality detection on chest radiography using game-like reinforcement mechanics. Acad Radiol.

[18] Isaacson G, Ianacone DC, Soliman AMS (2016). Ex vivo ovine model for suspension microlaryngoscopy training. J Laryngol Otol.

[19] Wouters P, van Oostendorp H (2013). A meta-analytic review of the role of instructional support in game-based learning. Comput Educ.

[20] Erhel S, Jamet E (2013). Digital game-based learning: Impact of instructions and feedback on motivation and learning effectiveness. Comput Educ.

[21] Toro-Troconis M, Kamat A, Partridge MR (2011). Design and development of a component-based system for virtual patients in the virtual world of second life®. J Emerg Techol Web Intell.

[22] Lorenzo-Alvarez R, Rudolphi-Solero T, Ruiz-Gomez MJ, Sendra-Portero F (2020). Game-based learning in virtual worlds: a multiuser online game for medical undergraduate radiology education within Second Life. Anat Sci Educ.

[23] Kye B, Han N, Kim E, Park Y, Jo S (2011). Educational applications of metaverse: possibilities and limitations. J Educ Eval Health Prof.

[24] Danforth DR, Procter M, Chen R, Johnson M, Heller R (2009). Development of virtual patient simulations for medical education. J Virtual Worlds Res.

[25] Creutzfeldt J, Hedman L, Felländer-Tsai L (2016). Cardiopulmonary resuscitation training by avatars: a qualitative study of medical students’ experiences using a multiplayer virtual world. JMIR Serious Games.

[26] Lee J, Kim H, Kim KH, Jung D, Jowsey T, Webster CS (2020). Effective virtual patient simulators for medical communication training: a systematic review. Med Educ.

[27] Kava BR, Andrade AD, Marcovich R, Idress T, Ruiz JG (2017). Communication skills assessment using human avatars: piloting a virtual world objective structured clinical examination. Urol Pract.

[28] Liaw SY, Carpio GAC, Lau Y, Tan SC, Lim WS, Goh PS (2018). Multiuser virtual worlds in healthcare education: A systematic review. Nurse Educ Today.

[29] Jivram T, Kavia S, Poulton E, Hernandez AS, Woodham LA, Poulton T (2021). The development of a virtual world problem-based learning tutorial and comparison with interactive text-based tutorials. Front Digit Health.

[30] Staziaki PV, Sarangi R, Parikh UN, Brooks JG, LeBedis CA, Shaffer K (2020). An objective structured clinical examination for medical student radiology clerkships: reproducibility study. JMIR Med Educ.

[31] Lorenzo-Alvarez R, Pavia-Molina J, Sendra-Portero F (2018). Exploring the potential of undergraduate radiology education in the virtual world Second Life with first-cycle and second-cycle Medical Students. Acad Radiol.

[32] (2022). Undergraduate radiology curriculum. The Royal College of Radiologists.

[33] (2025). Modern radiology in medical education: chapter 20 – emergency radiology. European Society of Radiology.

[34] Leschied JR, Knoepp US, Hoff CN (2013). Emergency radiology elective improves second-year medical students’ perceived confidence and knowledge of appropriate imaging utilization. Acad Radiol.

[35] Des Jarlais DC, Lyles C, Crepaz N, TREND Group (2004). Improving the reporting quality of nonrandomized evaluations of behavioral and public health interventions: the TREND statement. Am J Public Health.

[36] Paas FGWC, Van Merriënboer JJG (1994). Instructional control of cognitive load in the training of complex cognitive tasks. Educ Psychol Rev.

[37] Saldaña J (2013). The Coding Manual for Qualitative Researchers.

[38] Fromke EJ, Jordan SG, Awan OA (2022). Case-based learning: its importance in medical student education. Acad Radiol.

[39] Kolb AY, Kolb DA (2005). Learning styles and learning spaces: enhancing experiential learning in higher education. AMLE.

[40] Plass JL, Homer BD, Kinzer CK (2015). Foundations of game-based learning. Educ Psychol.

[41] Michaelsen LK, Sweet M (2008). The essential elements of team‐based learning. New Dir Teach Learn.

[42] Flowers MG, Aggarwal R (2014). Second Life, a novel simulation platform for the training of surgical residents. Expert Rev Med Devices.

[43] Aebersold M, Tschannen D, Stephens M, Anderson P, Lei X (2012). Second Life®: a new strategy in educating nursing students. Clin Simul Nurs.

[44] Deci EL, Ryan RM (2000). The “What” and “Why” of goal pursuits: human needs and the self-determination of behavior. Psychol Inq.

[45] Bers MU (2019). Coding as another language: a pedagogical approach for teaching computer science in early childhood. J Comput Educ.

[46] van Houten-Schat MA, Berkhout JJ, van Dijk N, Endedijk MD, Jaarsma ADC, Diemers AD (2018). Self-regulated learning in the clinical context: a systematic review. Med Educ.

[47] Smeby SS, Lillebo B, Slørdahl TS, Berntsen EM (2020). Express team-based learning (eTBL): a time-efficient TBL approach in neuroradiology. Acad Radiol.

[48] Van Gaalen AEJ, Jaarsma ADC, Georgiadis JR (2021). Medical students’ perceptions of play and learning: qualitative study with focus groups and thematic analysis. JMIR Serious Games.

[49] Andrade AD, Cifuentes P, Oliveira MC, Anam R, Roos BA, Ruiz JG (2011). Avatar-mediated home safety assessments: piloting a virtual objective structured clinical examination station. J Grad Med Educ.

[50] Sánchez E, Wouters P, van Oostendorp H (2017). Instructional Techniques to Facilitate Learning and Motivation of Serious Games Advances in Game-Based Learning.

[51] Ryan RM, Deci EL (2000). Self-determination theory and the facilitation of intrinsic motivation, social development, and well-being. Am Psychol.

[52] Gagné M, Deci EL (2005). Self‐determination theory and work motivation. J Organ Behavior.

[53] Featherstone M, Habgood J (2019). UniCraft: Exploring the impact of asynchronous multiplayer game elements in gamification. Int J Hum Comput Stud.

[54] Rudolphi-Solero T, Lorenzo-Alvarez R, Ruiz-Gomez MJ, Sendra-Portero F (2022). Impact of compulsory participation of medical students in a multiuser online game to learn radiological anatomy and radiological signs within the virtual world Second Life. Anat Sci Educ.

[55] Rudolphi-Solero T, Jimenez-Zayas A, Lorenzo-Alvarez R, Domínguez-Pinos D, Ruiz-Gomez MJ, Sendra-Portero F (2021). A team-based competition for undergraduate medical students to learn radiology within the virtual world Second Life. Insights Imaging.

[56] Pino-Postigo A, Domínguez-Pinos D, Lorenzo-Alvarez R, Pavía-Molina J, Ruiz-Gómez MJ, Sendra-Portero F (2023). Improving oral presentation skills for radiology residents through clinical session meetings in the virtual world Second Life. Int J Environ Res Public Health.

[57] Lorenzo-Alvarez R, Rudolphi-Solero T, Ruiz-Gomez MJ, Sendra-Portero F (2019). Medical student education for abdominal radiographs in a 3D virtual classroom versus traditional classroom: A randomized controlled trial. AJR Am J Roentgenol.

[58] Lewis PJ, Shaffer K (2005). Developing a national medical student curriculum in radiology. J Am Coll Radiol.

[59] Second Life system requirements. Second Life.

[60] de Oliveira Kubrusly Sobral JB, Lima DLF, Lima Rocha HA (2022). Active methodologies association with online learning fatigue among medical students. BMC Med Educ.

[61] Mollick ER, Rothbard N (2014). Mandatory Fun: Consent, Gamification and the Impact of Games at Work.

[62] Ghanbarzadeh R, Hossein Ghapanchi A (2020). Antecedents and consequences of user acceptance of three-dimensional virtual worlds in higher education. J Inf Technol Educ: Res.

[63] Ghanbarzadeh R, Ghapanchi AH (2023). Drivers of users’ embracement of 3D digital educational spaces in higher education: a qualitative approach. Tech Know Learn.

[64] García-Seoane JJ, Ramos-Rincón JM, Lara-Muñoz JP, el grupo de trabajo de la ECOE-CCS de la CNDFME, Miembros del grupo de trabajo de la ECOE-CCS de la CNDFME (por orden alfabético de Universidad) (2021). Changes in the Objective Structured Clinical Examination (OSCE) of University Schools of Medicine during COVID-19. Experience with a computer-based case simulation OSCE (CCS-OSCE). Rev Clin Esp.

