# Supplementary material for: Gamified Learning in a Virtual World for Undergraduate Emergency Radiology Education: Quasi-Experimental Study
Source: JMIR Med Educ. 2025 Aug 5;11:e68518. doi: 10.2196/68518 (PMC12324901; doi:10.2196/68518)
Supplement: Multimedia Appendix 1 [file mededu-v11-e68518-s001.pdf]

Appendix 1A. Presentation of the 16 clinical cases and the checklist used for their evaluation (0–10 points per case).

CASE 1 – Clinical situation

52-year-old man transferred to the emergency department after a sudden episode of intense headache, followed by a fall and loss of consciousness. Past medical history: Hypertension, type 2 diabetes mellitus, dyslipidemia, obesity, smoker. On arrival to the critical care unit, he presents with a Glasgow Coma Scale score of 8.

An imaging study is performed.

1) Describe the technique and pathological findings as accurately as possible.
2) Establish a differential diagnosis of possible pathological causes
3) What is your opinion on the prognosis?

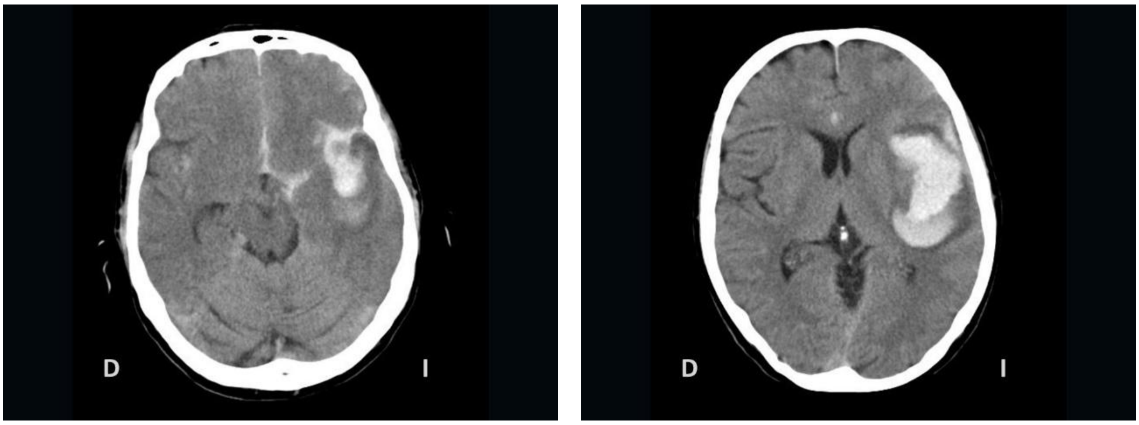

Two of 18 images available

CHECK LIST

1) Axial cranial CT – 1 point

1) Without contrast – 1 point

1) Bleeding/Hyperdensity in sulci – 1 point

1) Bleeding/Hyperdensity in cisterns/suprasellar cistern – 1 point

1) Intraparenchymal hematoma – 1 point

1) Mass effect/Compression of the lateral ventricle – 1 point

2) Hypertensive hematoma – 1 point

2) Rupture of AVM or aneurysm – 1 point

2) Hemorrhage associated with underlying tumor – 1 point

3) Poor/clinical deterioration given Glasgow score, SAH – 1 point

Appendix 1A. Presentation of the 16 clinical cases and the checklist used for their evaluation (0–10 points per case).

CASE 2 – Clinical situation

39-year-old woman transferred to the emergency department after a traffic accident while riding as a passenger on a motorcycle. She is conscious, oriented, and cooperative. She complains of abdominal and flank pain and presents with hematuria.

An imaging study is performed.

1) Describe the technique used and the pathological findings as accurately as possible.
2) What is your clinical judgment?
3) What would be your next step and why?

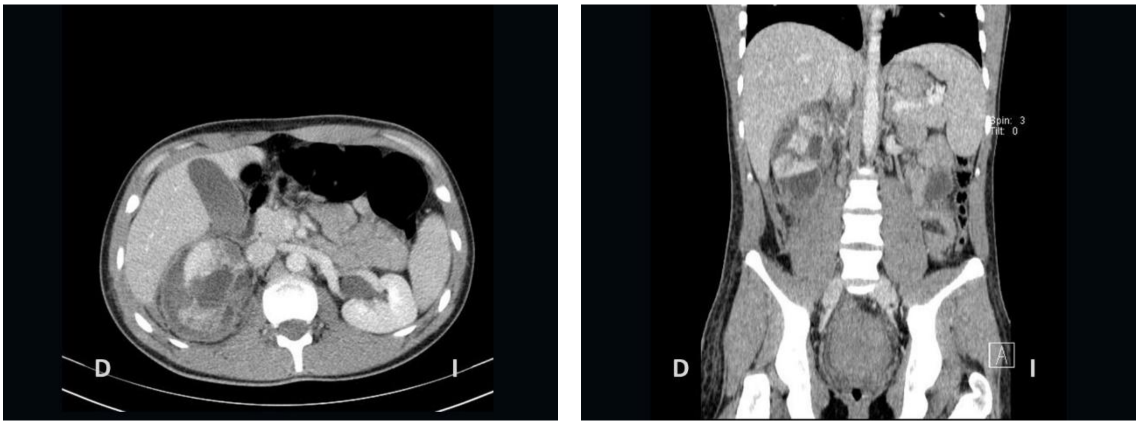

Two of 36 images available (two series of 18 each).

CHECK LIST

1) Axial and coronal abdominal CT – 1 point

1) With contrast – 1 point

1) Multiple hypodense lines/lacerations/renal fractures – 2 points

1) Peritoneal bleeding/hemoperitoneum – 1 point

2) Renal rupture/fracture/multiple lacerations – 2 points

2) Hemoperitoneum – 1 point

3) Urgent surgery (renal rupture) – 1 point

3) Hemodynamic support/fluid therapy/blood transfusion if needed – 1 point

Appendix 1A. Presentation of the 16 clinical cases and the checklist used for their evaluation (0–10 points per case).

CASE 3 – Clinical situation

65-year-old woman who presents to the emergency department with progressive shortness of breath, now occurring with minimal exertion. She reports weight loss over the past month. Past medical history: Smoker and dyslipidemic. Examination: Conscious, oriented, and cooperative. Mild respiratory effort noted. Global hypoventilation in the left lung. Oxygen saturation at 85%. Afebrile.

An imaging study is performed.

1) Describe the technique used and the pathological findings.
2) Establish a differential diagnosis and clinical suspicion
3) What additional tests would you request and why?

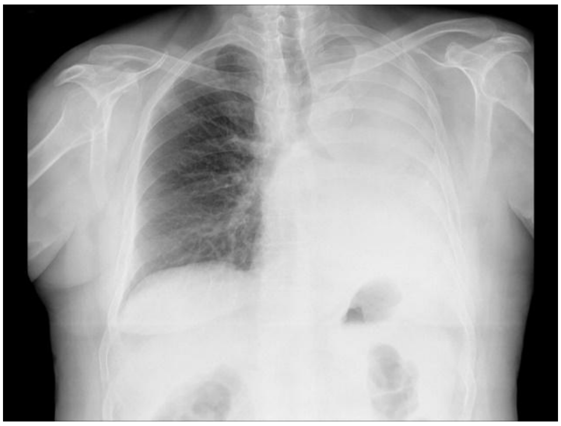

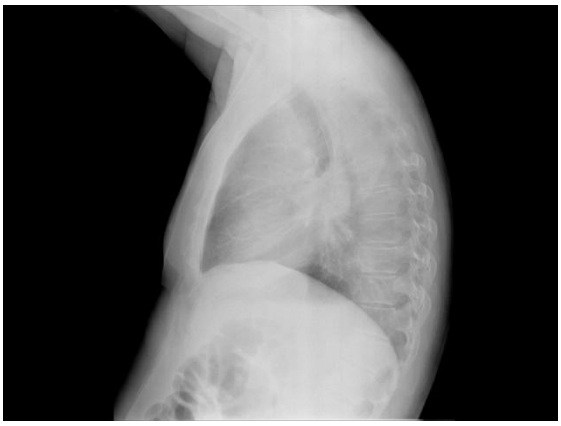

Two of 2 images available.

CHECK LIST

1) Plain chest X-ray PA view, well inspired, centered, and ventilated – 1 point

1) Complete left lung atelectasis – 1 point

1) Interrupted/amputated left main bronchus – 1 point

1) Central compressive pulmonary mass – 1 point

2) Atelectasis + effusion and left bronchial stenosis – 1 point

2) Central tumor – 2 points

3) Thoracic CT with IV contrast – 1 point

3) To characterize the tumor and its features – 1 point

3) Bronchoscopy/FNA of the lung mass – 1 point

Appendix 1A. Presentation of the 16 clinical cases and the checklist used for their evaluation (0–10 points per case).

CASE 4 – Clinical situation

79-year-old man transferred to the emergency department after his family found him speaking strangely, with a drooping mouth corner on the left and no strength in the right side of his body. On examination, the patient is somewhat stuporous but responds to pain, has incomprehensible speech, left facial palsy, and complete right-sided hemiplegia.

1) Describe the technique and pathological findings.
2) Provide clinical judgment and prognosis.
3) What is your immediate management proposal?

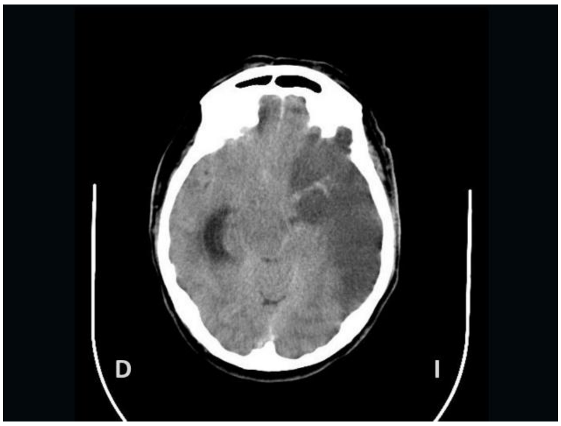

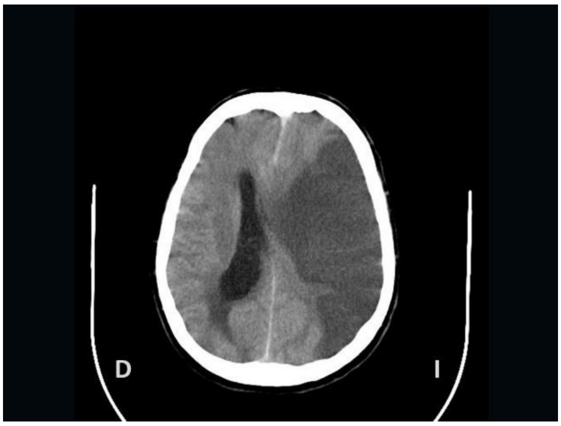

Two of 18 images available.

CHECK LIST

1) Axial cranial CT – 1 point

1) Without IV contrast – 1 point

1) Intraparenchymal hypodensity in the left cerebral hemisphere – 1 point

1) Involves frontal, parietal, occipital, temporal lobes – 1 point

1) Hemorrhage (laminar cortical necrosis) – 1 point

1) Mass effect/Midline shift or subtle left uncal/subfalcine herniation – 1 point

2) Ischemic lesion – 1 point

2) Acute – 1 point

2) Massive, evolving – 1 point

3) Hospital admission and clinical support – 1 point

Appendix 1A. Presentation of the 16 clinical cases and the checklist used for their evaluation (0–10 points per case).

CASE 5 – Clinical situation

73-year-old man with an episode of abdominal distension and intense pain, following several days of constipation. He is very agitated and presents with marked abdominal tympanism.

An imaging study is performed.

1) Describe the technique and pathological findings.
2) Provide clinical judgment and prognosis.
3) Immediate management proposal.

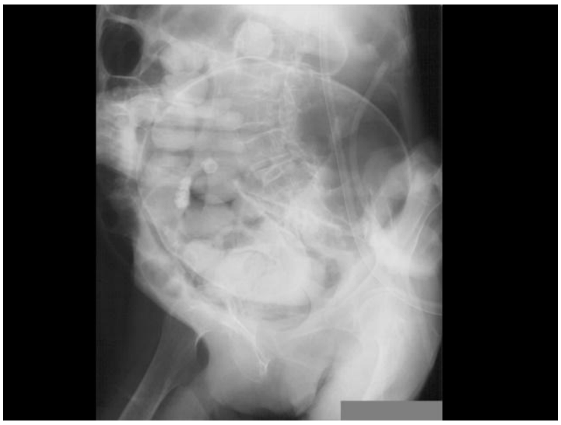

A single image available.

CHECK LIST

1) Plain abdominal X-ray – 1 point

1) Lateral view – 1 point

1) Intestinal loop dilation – 1 point

1) In large bowel – 2 points

1) Coffee-bean sign – 2 points

2) Sigmoid volvulus – 1 point

2) Poor prognosis, perforation present – 1 point

3) Urgent surgery – 1 point

Appendix 1A. Presentation of the 16 clinical cases and the checklist used for their evaluation (0–10 points per case).

CASE 6 – Clinical situation

55-year-old woman who presents to the emergency department with dull pain in the right upper quadrant, nausea, vomiting, and a fever of 38.5°C. Lab results: Notable for direct hyperbilirubinemia, elevated transaminases, CRP 89.2, and leukocytosis with a left shift. An imaging study is performed.

1) Describe as accurately as possible the technique used and the pathological findings.
2) Establish a differential diagnosis based on the findings and the clinical history.
3) What would be your proposal for the patient's immediate management?

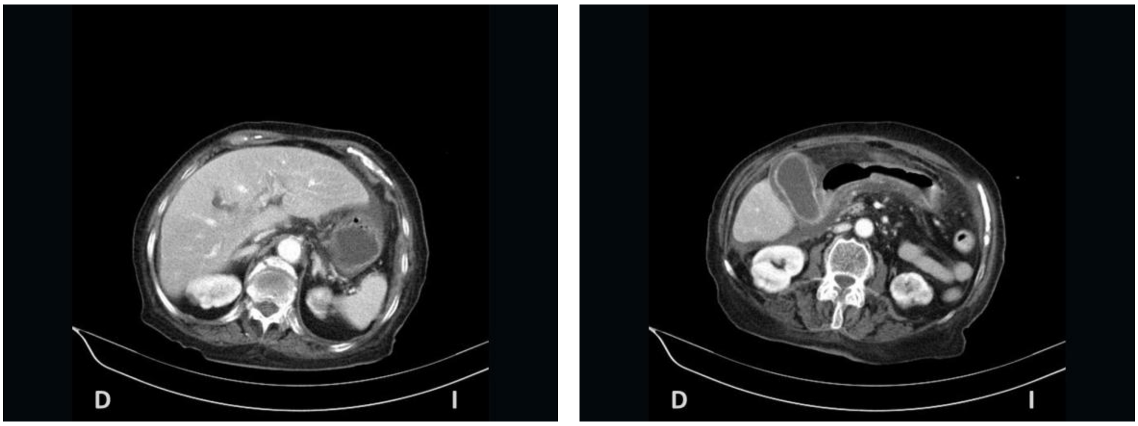

Two of 18 images available.

CHECK LIST

1) Abdominal CT – 1 point

1) With contrast – 1 point

1) Enlarged gallbladder – 1 point

1) Edematous gallbladder wall – 1 point

1) Increased fat attenuation – 1 point

1) Free fluid – 1 point

1) Bile duct dilation – 1 point

2) Acute cholecystitis – 1 point

3) Urgent surgery – 1 point

3) In-hospital antibiotics – 1 point

Appendix 1A. Presentation of the 16 clinical cases and the checklist used for their evaluation (0–10 points per case).

CASE 7 – Clinical situation

40-year-old man who presents to the emergency department after a traumatic brain injury (TBI) following a fall while riding a bicycle without a helmet. He has intense headache unresponsive to intravenous analgesics. His wife reports that he seems strange, like dazed or confused. An imaging study is performed.

1) Describe as accurately as possible the technique used and the pathological findings.
2) Establish a differential diagnosis based on the findings and the clinical history.
3) What would be your proposal for the patient's immediate management?

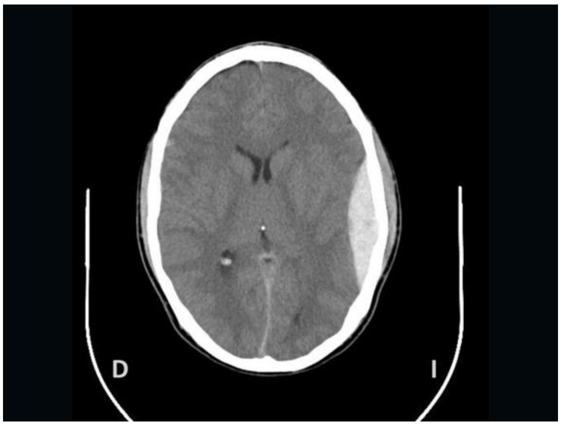

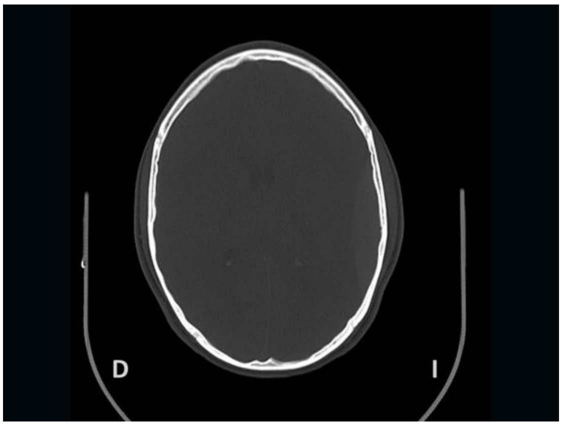

Two of 36 images available (two series of 18 each).

CHECK LIST

1) Axial cranial CT – 1 point

1) Without IV contrast – 1 point

1) Hyperdense lesion – 1 point

1) Biconvex – 1 point

1) Extra-axial – 2 points

1) Mass effect/Left lateral ventricle compression/slight subfalcine herniation – 1 point

1) Temporal skull fracture – 1 point

2) Acute epidural hematoma – 1 point

3) Urgent neurosurgical intervention/drainage – 1 point

Appendix 1A. Presentation of the 16 clinical cases and the checklist used for their evaluation (0–10 points per case).

CASE 8 – Clinical situation

59-year-old woman who presents to the emergency department with oppressive chest pain radiating to both sides, accompanied by significant shortness of breath. No acute ischemic changes on ECG. Blood pressure: 140/90.

A chest X-ray is performed.

1) Describe the radiological findings as accurately as possible.
2) What is your suspected diagnosis based on the findings and clinical history?
3) What test would you request immediately and why?

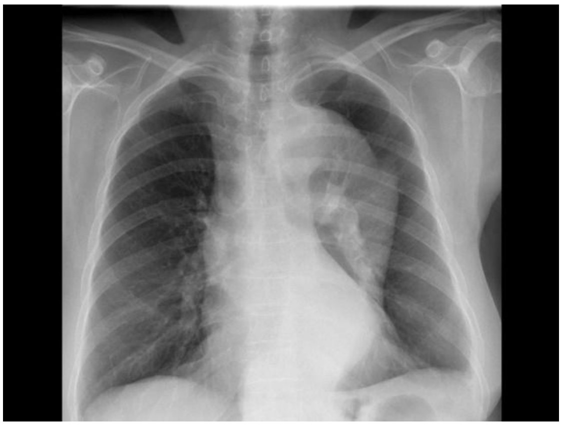

A single image available.

CHECK LIST

1) Plain chest X-ray PA view – 1 point

1) Well inspired, ventilated, and centered – 1 point

1) Aortic dilation – 1 point

1) Aortic knob (step-off sign) – 2 points

2) Aortic aneurysm with dissection – 2 points

3) Chest Angio-CT – 2 points

3) To assess dissection extent pre-surgery – 1 point

Appendix 1A. Presentation of the 16 clinical cases and the checklist used for their evaluation (0–10 points per case).

CASE 9 – Clinical situation

55-year-old man who presents to the emergency department with intense abdominal pain and loss of consciousness. On arrival he is hypotensive, heart rate 140, with marked skin and mucosal pallor, and absent pedal pulses.

1) Describe as accurately as possible the technique used and the pathological findings.
2) Establish a differential diagnosis based on the findings and the clinical history.
3) What would be your proposal for the patient's immediate management?

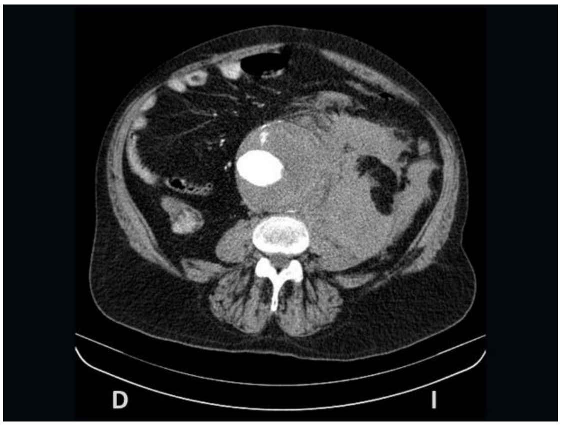

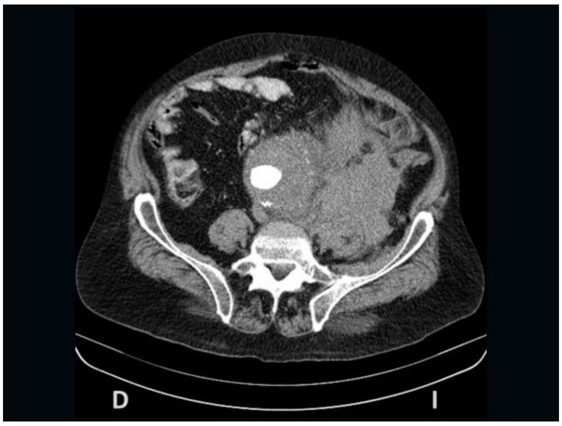

Two of 18 images available.

CHECK LIST

1) Abdominal CT – 1 point

1) With IV contrast – 1 point

1) Abdominal aortic aneurysm – 1 point

1) Rupture/discontinuity – 1 point

1) Hemoperitoneum – 1 point

2) Ruptured aortic aneurysm – 2 points

3) Urgent surgery – 1 point

3) Aortobiiliac endoprosthesis – 1 point

3) Hemodynamic support/transfusion – 1 point

Appendix 1A. Presentation of the 16 clinical cases and the checklist used for their evaluation (0–10 points per case).

CASE 10 – Clinical situation

58-year-old man, construction worker, who suffered a fall from a height of 2 meters while working, hitting his head. Glasgow Coma Scale score: 12–13. He reports severe headache and does not remember what happened.

1) Describe as accurately as possible the technique used and the pathological findings.
2) Establish a differential diagnosis based on the findings and the clinical history.
3) What would be your proposal for the patient's immediate management?

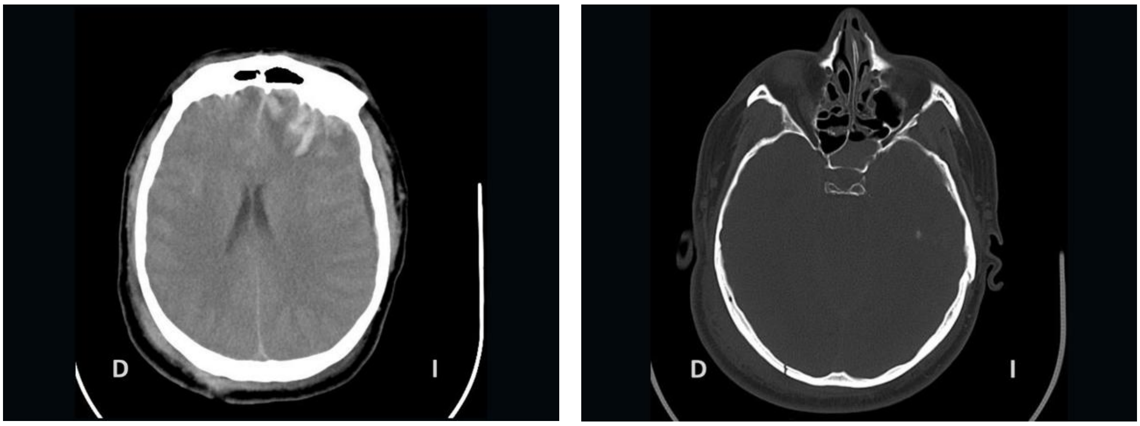

Two of 36 images available (two series of 18 each).

CHECK LIST

1) Cranial CT axial slices – 1 point

1) Without IV contrast – 1 point

1) Multiple contusions/intra-axial hemorrhages in right cerebellum, bilateral frontal – 1 point

1) Subdural hematoma (frontal, temporal left and cerebellar tentorium) – 1 point

1) Mass effect/left lateral ventricle compression – 1 point

1) Right maxillary hemosinus – 1 point

1) Occipital skull fracture – 1 point

2) Intra-axial hemorrhage/contusions and post-traumatic subdural hematoma – 1 point

3) Hospital admission, ventilatory/hemodynamic support – 1 point

3) Follow-up CT for potential subdural evacuation – 1 point

Appendix 1A. Presentation of the 16 clinical cases and the checklist used for their evaluation (0–10 points per case).

CASE 11 – Clinical situation

65-year-old man who presents to the emergency department with high fever lasting several days. He also reports fatigue and rust-colored sputum. Marked leukocytosis with neutrophilia and elevated CRP.

Chest X-rays are performed.

1) Describe the radiographic findings as accurately as possible.
2) Establish a differential diagnosis based on the findings and the clinical history.
3) What would be your proposal for the patient's immediate management?

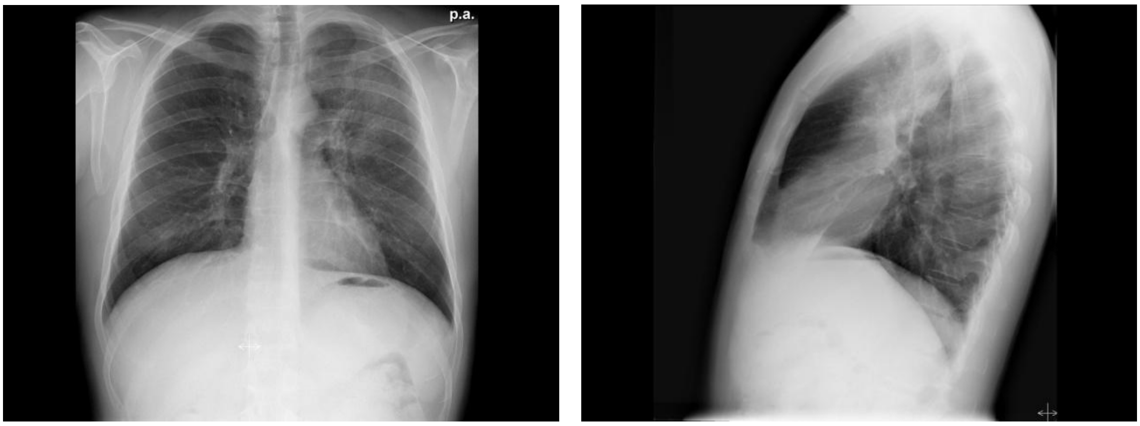

Two of 2 images available.

CHECK LIST

1) Chest X-ray PA and lateral – 1 point

1) Well inspired, centered, ventilated – 1 point

1) Increased density in left upper lobe – 2 points

1) Air bronchogram – 1 point

2) Bacterial pneumonia – 2 points

2) Superinfected atypical pneumonia – 1 point

3) Antibiotics – 1 point

3) Outpatient management due to good clinical status – 1 point

Appendix 1A. Presentation of the 16 clinical cases and the checklist used for their evaluation (0–10 points per case).

CASE 12 – Clinical situation

60-year-old man with a history of chronic constipation who presents to the emergency department with left iliac fossa pain and low-grade fever. He has mild leukocytosis with left shift and a CRP of 62.

Abdominal ultrasound is inconclusive, so an abdominopelvic CT scan is performed.

1) Describe the radiological findings as accurately as possible.
2) What is your differential diagnosis based on the findings and clinical history?
3) What would be your proposal for the patient's immediate management?

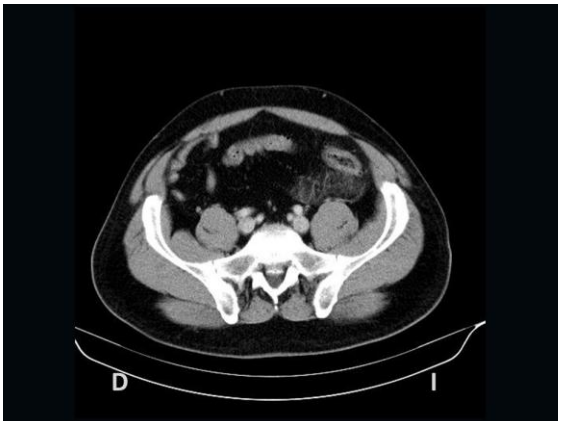

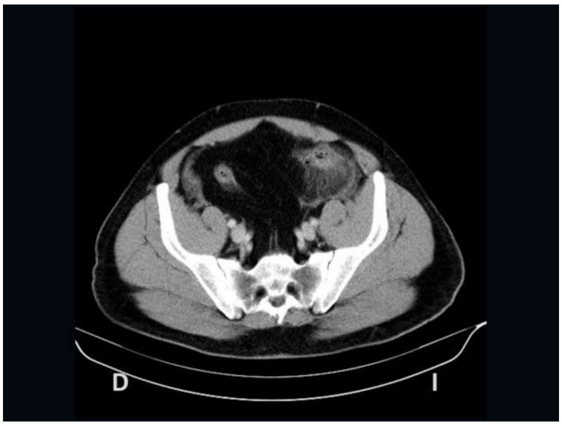

Two of 18 images available.

CHECK LIST

1) Sigmoid colon wall thickening – 1 point

1) Area of diverticulosis – 1 point

1) Increased pericolic fat attenuation – 1 point

1) Enhancement of one diverticulum – 1 point

1) Small amount of pericolic fluid – 1 point

2) Acute diverticulitis – 2 points

2) Infectious colitis in diverticulosis area – 1 point

3) Hospital admission – 1 point

3) Antibiotics – 1 point

Appendix 1A. Presentation of the 16 clinical cases and the checklist used for their evaluation (0–10 points per case).

CASE 13 – Clinical situation

62-year-old man who presents to the emergency department reporting shortness of breath, fatigue, joint pain, muscle aches, loss of smell, and a recorded fever of 38.5°C.

Chest X-rays are performed.

1) Describe the radiographic findings as accurately as possible.
2) Establish a differential diagnosis based on the findings and the clinical history.
3) What would be your proposal for the patient's immediate management?

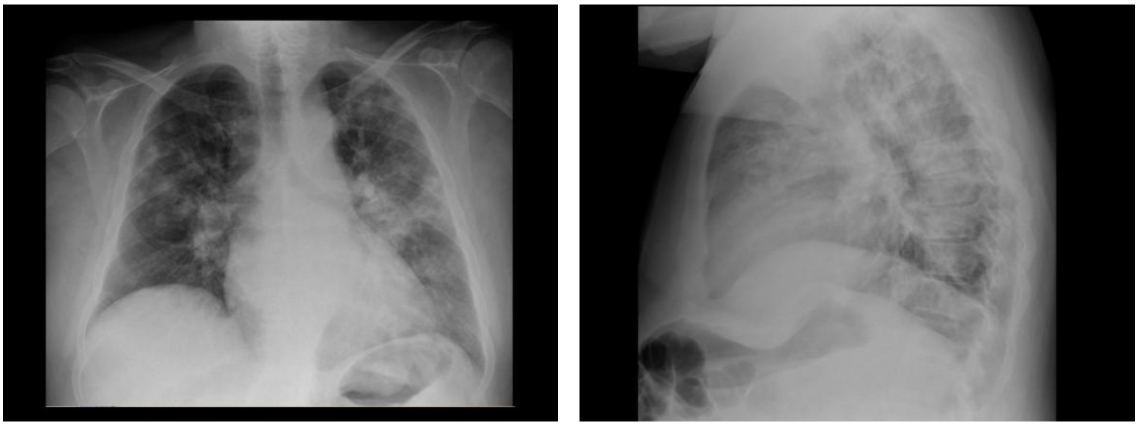

Two of 2 images available.

CHECK LIST

1) Multiple interstitial nodular infiltrates – 2 points

1) Bilateral – 1 point

2) Worsening viral infection – 1 point

2) COVID-19 – 3 points

2) Atypical mycobacterial pneumonia – 1 point

3) Hospital admission – 1 point

3) Antipyretics and analgesics – 1 point

Appendix 1A. Presentation of the 16 clinical cases and the checklist used for their evaluation (0–10 points per case).

CASE 14 – Clinical situation

55-year-old woman who reports having a headache for several days that has not improved with usual analgesics. Her husband insisted on bringing her to the emergency department because he finds her “a bit off.” On examination, she is conscious and oriented, but shows difficulty understanding language. Lab results are normal. She is afebrile.

1) Describe as accurately as possible the technique used and the pathological findings.
2) Establish a differential diagnosis based on the findings and the clinical history.
3) What would be your proposal for the patient’s immediate management?

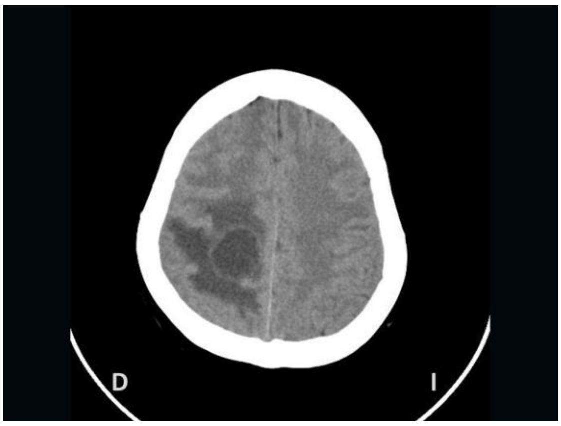

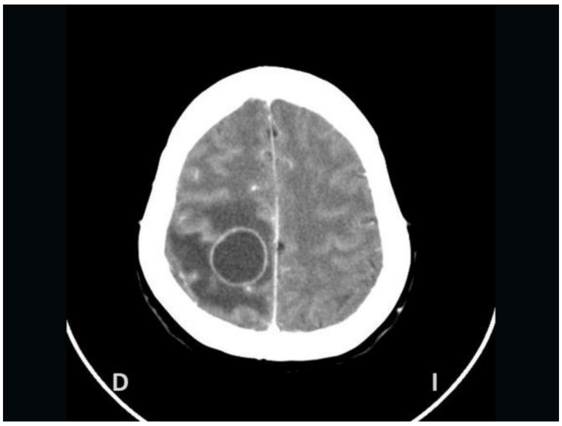

Two of 36 images available (two series of 18 each).

CHECK LIST

1) Cranial CT with and without IV contrast – 1 point

1) Rounded mass/lesion – 1 point

1) Hypodense, cystic/necrotic – 1 point

1) Right parietal lobe – 1 point

1) Perilesional edema – 1 point

2) Brain metastasis – 1 point

2) Primary brain tumor – 1 point

2) Toxoplasmosis – 1 point

2) Brain abscess – 1 point

3) Hospital admission for evaluation (MRI with IV contrast) – 1 point

Appendix 1A. Presentation of the 16 clinical cases and the checklist used for their evaluation (0–10 points per case).

CASE 15 – Clinical situation

49-year-old man reporting pain in the left renal fossa for one week, with decreased urine output. Conscious, oriented, and afebrile on examination. Creatinine is 2.5, slightly elevated compared to previous normal levels. Positive psoas sign. An ultrasound is performed by the radiologist, who then proceeds directly to a CT scan.

1) Describe as accurately as possible the technique used and the pathological findings.
2) Establish a differential diagnosis based on the imaging findings and clinical history.
3) What would be your proposal for the patient's immediate management?

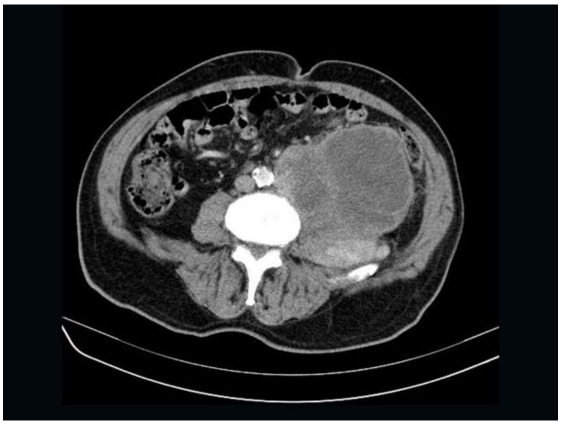
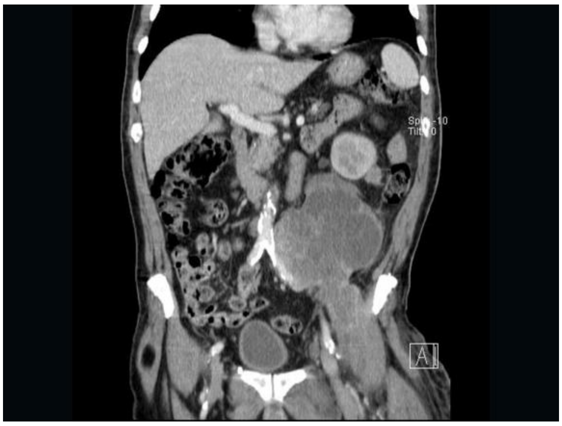

Two of 36 images available (two series of 18 each).

CHECK LIST

1) Abdominal CT with IV contrast, axial and coronal – 1 point

1) Left ureteropelvicalyceal dilatation due to obstruction – 2 points

1) Large mass – 1 point

1) Heterogeneous – 1 point

1) Retroperitoneal – 2 points

2) Soft tissue neoplastic process – 1 point

2) Probable sarcoma – 1 point

3) Hospital admission and image-guided biopsy (ultrasound/CT) – 1 point

Appendix 1A. Presentation of the 16 clinical cases and the checklist used for their evaluation (0–10 points per case).

CASE 16 – Clinical situation

76-year-old man, long-time ex-smoker. He presents to the emergency department with generalized chest pain that prevents him from sleeping. Reports weight loss in recent months. O_2 saturation is 90%. Lab results: Elevated alkaline phosphatase, all other values normal. A chest X-ray is performed.

1) Describe the pathological findings as accurately as possible.
2) Establish a differential diagnosis based on the imaging findings and clinical history.
3) What would be your proposal for the patient's immediate management?

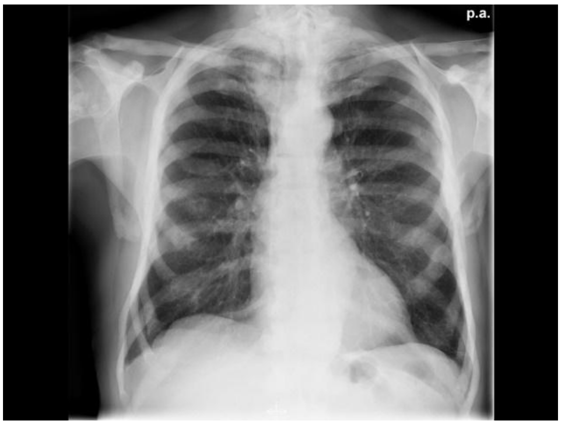

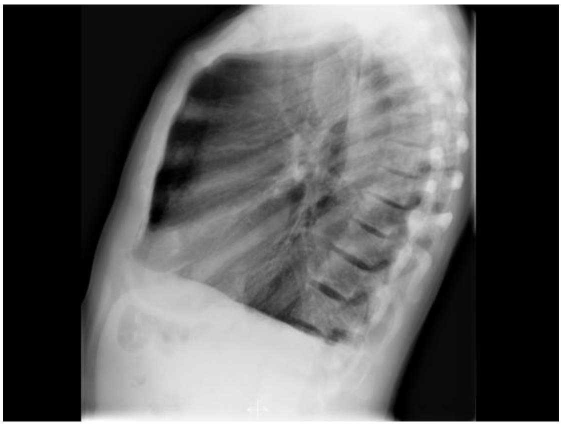

Two of 2 images available.

CHECK LIST

1) Costal hyperdensity – 1 point

1) Vertebral hyperdensity – 1 point

1) Clavicular/scapular hyperdensity – 1 point

2) Bone metastases – 2 points

2) Prostate tumor – 2 points

3) Prostate MRI with IV contrast – 1 point

3) Consider urologic prostate biopsy – 1 point

3) Thoracoabdominal staging CT – 1 point

Appendix 1B. Presentation of the seminar exam cases, and the checklist used for their correction (24-item checklist).

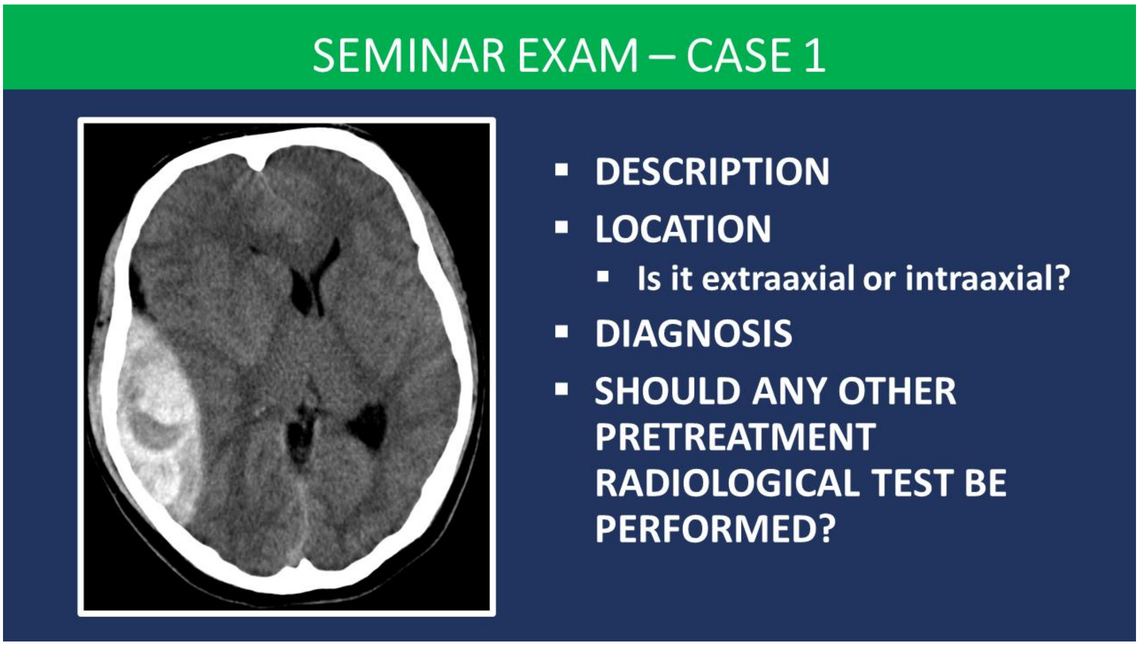

CHECK LIST CASE 1

DESCRIPTION

#1 Biconvex lesion

#2 Hyperdense

#3 Hyperacute/Heterogeneous

#4 Midline displacement

LOCATION

#5 Extraaxial

DIAGNOSIS

#6 Acute epidural hematoma

#7 Subfalcine hernia due to mass effect

SHOULD ANY OTHER RADIOLOGICAL PRETREATMENT TEST BE PERFORMED?

#8 No further imaging tests are necessary.

#9 Urgent neurosurgical drainage

Appendix 1B. Presentation of the seminar exam cases, and the checklist used for their correction (24-item checklist).

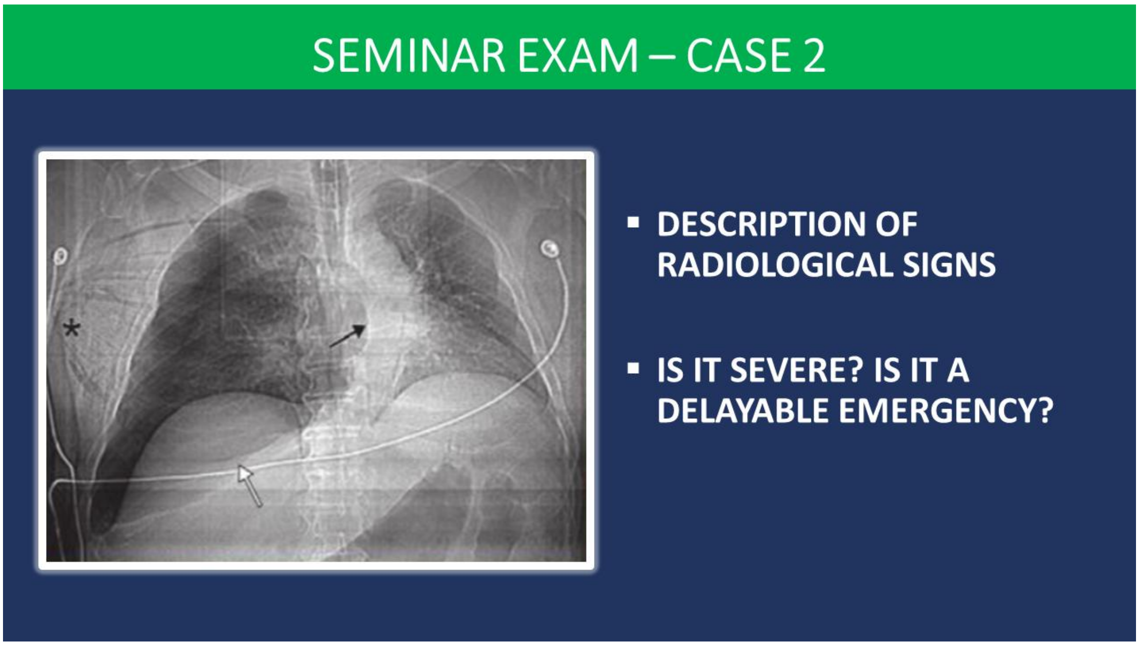

CHECKLIST CASE 2

DESCRIPTION OF RADIOLOGICAL SIGNS

#10 Mediastinal and tracheal displacement

#11 Displacement of the pleuroazygoesophageal line

#12 Deep costophrenic sinus sign

#13 Tracheal intubation

#14 Subcutaneous emphysema

#15 Hyperclarity of the right upper quadrant/diaphragmatic flattening

#16 Widening of intercostal spaces.

#17 Ipsilateral lung collapse

IS IT SEVERE? IS IT A DELAYABLE EMERGENCY?

#18 Non-delayable medical emergency due to compromised cardiac diastolic filling

#19 Urgent placement of chest drain

Appendix 1B. Presentation of the seminar exam cases, and the checklist used for their correction (24-item checklist).

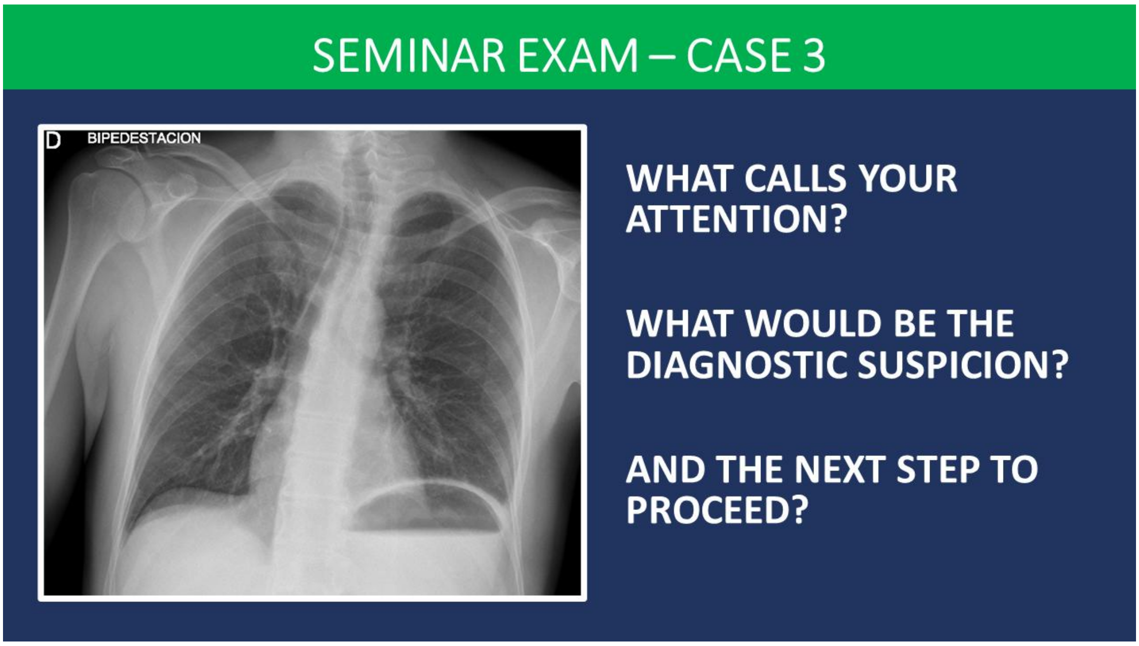

CHECK LIST CASE 3

WHAT CALLS YOUR ATTENTION?

#20 Extraluminal air in left hypochondrium/Pneumoperitoneum

WHAT WOULD BE THE DIAGNOSTIC SUSPICION?

#21 Abdominal perforation / hollow viscus

AND THE NEXT STEP TO PROCEED?

#22 AngioTC

#23 Intravenous contrast

#24 Call surgery
