# Supplementary material for: Gamified Learning in a Virtual World for Undergraduate Emergency Radiology Education: Quasi-Experimental Study
Source: JMIR Med Educ. 2025 Aug 5;11:e68518. doi: 10.2196/68518 (PMC12324901; doi:10.2196/68518)
Supplement: Multimedia Appendix 2 [file mededu-v11-e68518-s002.pdf]

QUESTIONNAIRE **Rainbow-Game** 2021

Did you know Second Life before this experience (Y/N).. ☐

Game rating: Scale from 1 to 5.

Please, complete the following questions in the box, scoring from 1 to 5

1) Totally disagree
2) Disagree
3) Neither agree nor disagree
4) Agree
5) Totally agree

Rating: **scale from 1 to 5**

Your computer meets the requirements to work in Second Life without problems. ☐
Your internet connection allows you to work in Second Life without problems ...... ☐
The environment of the OSCE rooms seemed attractive to you........................... ☐
The design of the competition seemed correct to you .... ☐
The information about the competition was adequate .... ☐
The selection of the OSCE cases seemed appropriate for your training .... ☐
The contents of the seminar seemed adequate to you .... ☐
Your participation in the seminar was very active .... ☐
You worked as a team in this experience .... ☐
You had fun in this experience .... ☐
Learning Radiology in Second Life seems interesting to you .... ☐
Playing and competing in Second Life you learn better .... ☐
You would participate in another Second Life experience when you are a resident .. ☐

Mental effort: Scale from 1 to 9

Please answer from 1 to 9 how much mental effort it costs you to perform the following tasks:

1) 1) Very, very low mental effort
2) Very low mental effort
3) Low mental effort
4) Somewhat low mental effort
5) Neither high nor low mental effort
6) Somewhat high mental effort
7) High mental effort
8) Very high mental effort
9) Very, very high mental effort

Mental effort: scale form 1 to 9

Moving around in Second Life.. ☐
Communicate by written chat.. ☐
Communicate by voice .... ☐
Edit and dress your avatar .... ☐
Solve the proposed cases in the OSCE-RX room .... ☐
Follow the development of the seminar in Second Life .... ☐

- *This questionnaire collects data with the sole objective of carrying out a study to evaluate the educational innovation experience to which it refers. The information collected will not be used for any other purpose.*
- *• All data collected in this questionnaire will be recorded anonymously, and kept strictly in accordance with current legislation (Organic Law 3/2018, of December 5, on the Protection of Personal Data and guarantee of digital rights).*
- *• By completing and sending it you confirm your consent to participate in the study.*

QUESTIONNAIRE **Rainbow-Game** 2021

Important!: Rating from 0 to 10

Para finalizar, por favor califica dentro del boxo los siguientes aspectos, puntuando de 0 a 10

Rating from 0 to 10

Overall experience .... ☐

Organization of the project .... ☐

Environment of the OSCE rooms .... ☐

OSCE cases .... ☐

The virtual seminar .... ☐

The teachers .... ☐

The utility for your training .... ☐

Interaction with peers .... ☐

Connectivity to Second Life... ☐

Open comments

If you want to add anything else, please use the box below.

- *This questionnaire collects data with the sole objective of carrying out a study to evaluate the educational innovation experience to which it refers. The information collected will not be used for any other purpose.*
- *• All data collected in this questionnaire will be recorded anonymously, and kept strictly in accordance with current legislation (Organic Law 3/2018, of December 5, on the Protection of Personal Data and guarantee of digital rights).*
- *• By completing and sending it you confirm your consent to participate in the study.*
